# Contextual factors and mechanisms that influence sustainability: a realist evaluation of two scaled, multi-component interventions

**DOI:** 10.1186/s12913-021-07214-5

**Published:** 2021-11-04

**Authors:** Rachel Flynn, Kelly Mrklas, Alyson Campbell, Tracy Wasylak, Shannon D. Scott

**Affiliations:** 1 Faculty of Nursing, Level 3, Edmonton Clinic Health Academy, University of Alberta, 11405 87 Avenue, Alberta T6G 1C9 Edmonton, Canada; 2 Strategic Clinical Networks™, Provincial Clinical Excellence, Alberta Health Services, Calgary, Canada; 3 Department of Community Health Sciences, Cumming School of Medicine, University of Calgary, T2N 4N1 Calgary, Canada; 4 Faculty of Nursing, University of Calgary, T2N 4V8 Alberta, Canada

**Keywords:** Sustainability, Strategic clinical networks, Quality improvement, Realist evaluation

## Abstract

**Background:**

In 2012, Alberta Health Services created Strategic Clinical Networks^TM^ (SCNs) to develop and implement evidence-informed, clinician-led and team-delivered health system improvement in Alberta, Canada. SCNs have had several provincial successes in improving health outcomes. Little research has been done on the sustainability of these evidence-based implementation efforts.

**Methods:**

We conducted a qualitative realist evaluation using a case study approach to identify and explain the contextual factors and mechanisms perceived to influence the sustainability of two provincial SCN evidence-based interventions, a delirium intervention for Critical Care and an Appropriate Use of Antipsychotics (AUA) intervention for Senior’s Health. The context (C) + mechanism (M) = outcome (O) configurations (CMOcs) heuristic guided our research.

**Results:**

We conducted thirty realist interviews in two cases and found four important strategies that facilitated sustainability: Learning collaboratives, audit & feedback, the informal leadership role, and patient stories. These strategies triggered certain mechanisms such as sense-making, understanding value and impact of the intervention, empowerment, and motivation that increased the likelihood of sustainability. For example, informal leaders were often hands-on and influential to front-line staff. Learning collaboratives broke down professional and organizational silos and encouraged collective sharing and learning, motivating participants to continue with the intervention. Continual audit-feedback interventions motivated participants to want to perform and improve on a long-term basis, increasing the likelihood of sustainability of the two multi-component interventions. Patient stories demonstrated the interventions’ impact on patient outcomes, motivating staff to want to continue doing the intervention, and increasing the likelihood of its sustainability.

**Conclusions:**

This research contributes to the field of implementation science, providing evidence on key strategies for sustainability and the underlying causal mechanisms of these strategies that increases the likelihood of sustainability. Identifying causal mechanisms provides evidence on the processes by which implementation strategies operate and lead to sustainability. Future work is needed to evaluate the impact of informal leadership, learning collaboratives, audit-feedback, and patient stories as strategies for sustainability, to generate better guidance on planning sustainable improvements with long term impact.

**Supplementary Information:**

The online version contains supplementary material available at 10.1186/s12913-021-07214-5.

## Background

It is well known that sustainability planning and processes are required well in advance of the implementation of evidence-based interventions (EBIs) for healthcare improvement [1]. Sustainability research is both fundamental to the field of implementation science and critical to the long-term viability of a publicly funded healthcare system [2–5]. Sustainability is comprised of a program, clinical intervention, and implementation strategies, including individual behavior change (e.g., clinician, patient) that continue to be delivered and are maintained after a defined period of time; during which the program and individual behavior change may evolve or adapt while continuing to produce benefits for individuals/systems [6].

Recent research on the sustainability of EBIs in healthcare, has identified key determinants for sustainability and theoretical approaches used to assess, plan, execute or evaluate sustainability [7–9]. Despite growing interest, the understanding of how to sustain the use of EBIs in healthcare remains relatively unexplored [3, 9]. Furthermore, little research has examined the causal mechanisms that influence the sustainability of such interventions. Mechanisms from an implementation science lens have been articulated as a process or event through which an implementation strategy operates to affect desired implementation outcomes. From a realist lens, mechanisms are the combination of resources (intended and unintended) offered by a social program under study (e.g., intervention) and the response to those resources (cognitive, emotional, motivational reasoning etc.) by stakeholders [10]. With this view, intervention resources are implemented in a context (e.g., implementation strategies), in a way that potentially alters the response and reasoning of stakeholders, changing their behavior, which leads to outcomes [11]. Mechanisms will only activate under the right contextual conditions. Mechanisms offer causal pathways explaining how strategies operate under certain contexts to achieve desired outcomes, such as sustainment of EBIs [12]. It is important to identify and explain the causal mechanisms for the sustainability of EBIs in healthcare to identify strategies that are most effective to enhance sustainment [13].

### Research aim

The aim of our study was to identify and explain the contextual factors and causal mechanisms that enabled or hindered the sustainability of two, large-scale, system-wide EBIs implemented across the Strategic Clinical Networks™, of the Alberta health system in Canada [14].

### Research context: strategic clinical networks, Alberta Health Services

The past decade marked a period of health system transformation in Alberta, as Canada’s first province-wide, fully integrated health system in 2008. One key objective of this integrated system is to embed evidence into healthcare practice to continuously improve health outcomes and health service delivery, ensuring high quality care and value for every Albertan. To support these objectives Alberta Health Services created Strategic Clinical Networks™ (SCNs) in 2012. SCNs comprise multi-stakeholder teams (e.g., patients, leaders and managers, clinicians, and researchers) that work collaboratively to identify care gaps and implement evidence-based interventions that improve health outcomes and health service delivery [15, 16]. Clinical healthcare networks, like SCNs, are intended to break down professional, organizational, and geographical boundaries by bringing multi-stakeholder groups together to co-design evidence-based interventions aimed to improve health care delivery and outcomes [17]. SCNs are embedded in Alberta Health Services (AHS), Canada’s first province-wide health care system serving 4.3 M people [18]. Currently, there are 11 SCNs and 5 Integrated Provincial Programs across Alberta, each with a specific scope and mandate, focused on various areas of health (i.e. cancer), areas of care (i.e. emergency care), provincial programs (i.e. senior’s health), specific populations (i.e. maternal, newborn, child and youth health) or spanning multiple disease areas (i.e. diabetes, obesity, nutrition) [15].

Previous research on SCNs have focused on implementation [19, 20], cost analysis [21, 22], or specific interventions [23, 24]. However, while these EBIs themselves have been evaluated, no studies to date have explicitly examined sustainability.

As SCNs mature and continue to embed evidence into practice through province wide implementation efforts, learning to spread and scale these interventions and to ensure sustainability is critical [25, 26]. Failure to sustain effective EBIs poses significant risks to individuals, healthcare systems, funding systems, and communities [27]. Recognizing and explaining key contextual factors and causal mechanisms that have hindered and facilitated SCN EBIs sustainability will contribute to systematic and comprehensive sustainability planning, design, and implementation. This realist evaluation case study examines two multi-component EBIs that have been spread and scaled across Alberta (Case A, Case B), providing an opportunity to better understand contextual factors and mechanisms that influence sustainability at scale.

### Strategic clinical network case selection

We purposefully selected two scaled, evidence-based, multi-component interventions based on (a) their maturity, (b) scale of implementation (province wide), (c) demonstration of improved outcomes and impact and, (d) context variation (community and acute healthcare). We defined a ‘case’ as an intervention that was evidence-based, had been formally implemented by the SCNs either within Alberta Health Services and /or with partner organizations. Case A is the Intensive Care Unit (ICU) Delirium intervention implemented at scale from 2016 to 19 across all 22 ICUs in Alberta. Case B is the Appropriate Use of Antipsychotics (AUA) implemented in two different sectors, long-term care (LTC, 170 sites) and designated supportive living (DSL, 140 sites). The AUA intervention was first piloted in 2013-14 in 11 early adopter sites and was spread provincially during 2014-15 to 170 LTC sites (both public and private); DSL implementation occurred from 2016 to 18 in 140 spaces both public and private settings (see additional file 1. for case descriptions).

## Methods

### Realist evaluation

We conducted a realist evaluation [10] using an explanatory case study research design [28] to study contextual factors and causal mechanisms that enabled or hindered the sustainability of two provincially scaled and spread multi-component EBIs or “cases”. Realist evaluation unpacks and explains the possible causes and contextual factors of change by examining “what works for whom, under what circumstances, and why?”, rather than merely assessing “does it work?” [10]. We followed the realist heuristic context (C) + mechanism (M) = outcome (O) configuration, whereby an intervention works or not (O), (CMOcs) because of the action of some underlying mechanism (M), which only comes into operation in particular contexts (C) [10, 29]. The realist terms used in this evaluation are provided in Table 1.

**Table 1:** Realist terms

Context	Context can be defined as all factors that are not part of the program or intervention itself, the “backdrop” to implementation, yet does interact, influence, modify, facilitate or hinder the intervention and its effectiveness [30].
Mechanisms	Mechanisms are the combination of resources (intended and unintended) offered by a social program under study (EBI: delirium, AUA) and the response to those resources (cognitive, emotional, motivational reasoning etc.) by stakeholders [10]. Mechanisms will only activate in the right conditions (contexts).
Outcomes	Outcomes are a result of a program firing multiple mechanisms which have different effects on different subjects in different situations, and so produce multiple outcomes. Realist evaluators examine outcome patterns in a theory testing role. Outcomes are analyzed to discover if conjectured mechanism/context theories are confirmed [10] (p. 217).
Context-mechanism-outcome configuration (CMOc)	CMO configuring is a heuristic used to generate causative explanations about outcomes in the observed data. A CMO configuration may be about the whole program or only to certain aspects. One CMO may be embedded in another or configured in a series (ripple effect in which the outcome of one CMO becomes the context for the next in the chain of implementation steps). Configuring CMOs is a basis for generating and/ or refining the theory that becomes the final product of the review [31] (p.3)

We followed the realist cycle of theory hypothesis generation, observation and specification [10] according to realist terms previously detailed [32]. We followed the Realist and Meta-narrative Evidence Synthesis: Evolving Standards (RAMESES) II reporting standards and SQUIRE 2.0 checklist [33, 34] (additional files 2 & 3).

### Initial program theory development

**Fig. 1:**
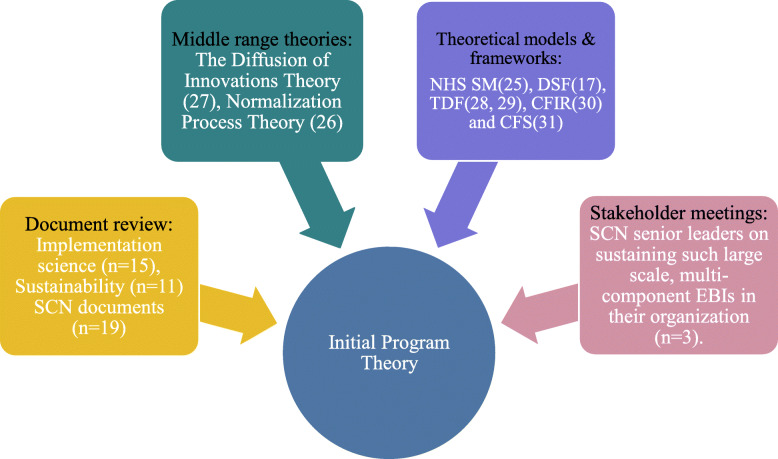
Evidence sources used to inform initial program theory development

Following the realist evaluation cycle, we first developed an initial program theory (IPT) to hypothesize how, why, for whom and under what contexts we expected these EBIs to be sustained. The sources used to inform our IPT are depicted in Fig. 1.

The first step in our IPT development was to review key implementation science (*n *= 15), sustainability (*n *= 11) and SCN documents (*n *= 19), including the identification of relevant theoretical links between implementation and sustainability. The National Health Services Sustainability Model [35], Dynamic Sustainability Framework [26] and Normalization Process Theory [36] were used to identify key contextual factors and mechanisms that influenced the likelihood of sustainability. The Diffusion of Innovations [37] theory was applied to help understand key characteristics that influence successful adoption. The Theoretical Domains Framework [38, 39] provided a validated way to link elements that influenced implementation, to a broad range of behavioral theories. Similarly, the Consolidated Framework for Implementation Research [40] and the Consolidated Framework for Sustainability [7] were used to make sense of diverse factors that influence implementation and potentially sustainability including intervention, contextual, individual and implementation process characteristics.

**Fig. 2:**
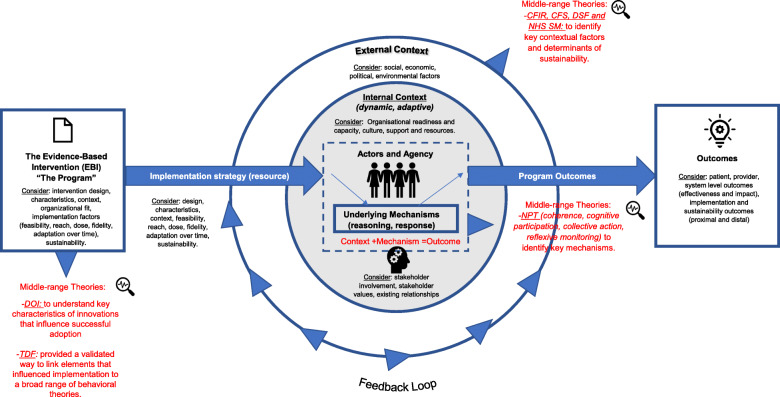
Visual representation of initial program theory

Second, we conducted key stakeholder meetings with three senior leaders from different SCNs, to explore their perspectives and experiences on sustaining such large scale, multi-component interventions in their organization. We used meeting notes to supplement information gathered from key documents. Information from our key stakeholder meetings and key documents informed the initial 64 CMOcs. Our team iteratively refined and thematically organized these CMOcs, yielding a final set of ten CMOcs. The IPT and ten CMOcs are provided in additional file 4, with a visual representation of our IPT provided in Fig. 2. We subsequently tested and refined these 10 CMOcs through realist interviews with multi-disciplinary healthcare providers (HCPs) involved in the two purposefully selected cases.

### Ethics approval

 Ethics approval for this study was granted by the University of Alberta Health Research Ethics Board (Pro0096202). Institutional approval was provided by Alberta Health Services Northern Alberta Clinical Trials and Research Centre.

### Recruitment and data collection

We purposefully selected interview participants involved with the implementation of each intervention across different levels of the healthcare system (i.e., front line staff, middle management, and senior management) and geographically across the province. This diverse selection of participants allowed for rich discussion on the similar and different features between implementation and sustainability from different perspectives. We contacted potential study participants through an open letter of invitation circulated to staff by Alberta Health Services leaders. Interested participants were invited to voluntarily contact the research assistant at their convenience for more information.

We conducted qualitative realist interviews using a semi-structured interview guide to test and further refine our initial program theory and explore new emerging CMOcs. Interviews explored participants’ perceptions of each intervention, implementation and sustainability processes, as well as the contextual factors and mechanisms that enabled or hindered sustainability. Our interview guide was informed by our IPT. We distinguished between implementation and sustainability in order to test our program theory and to unpack CMOcs relevant to sustainability. We applied the realist interviewing technique of the “teacher-learner cycle” where the interviewer presents the program theory to participants and gives them an opportunity to confirm, refute or refine the theory [41]. All interviews were conducted by telephone by the research assistant (AC), audio recorded and transcribed.

### Data analysis

**Fig. 3:**
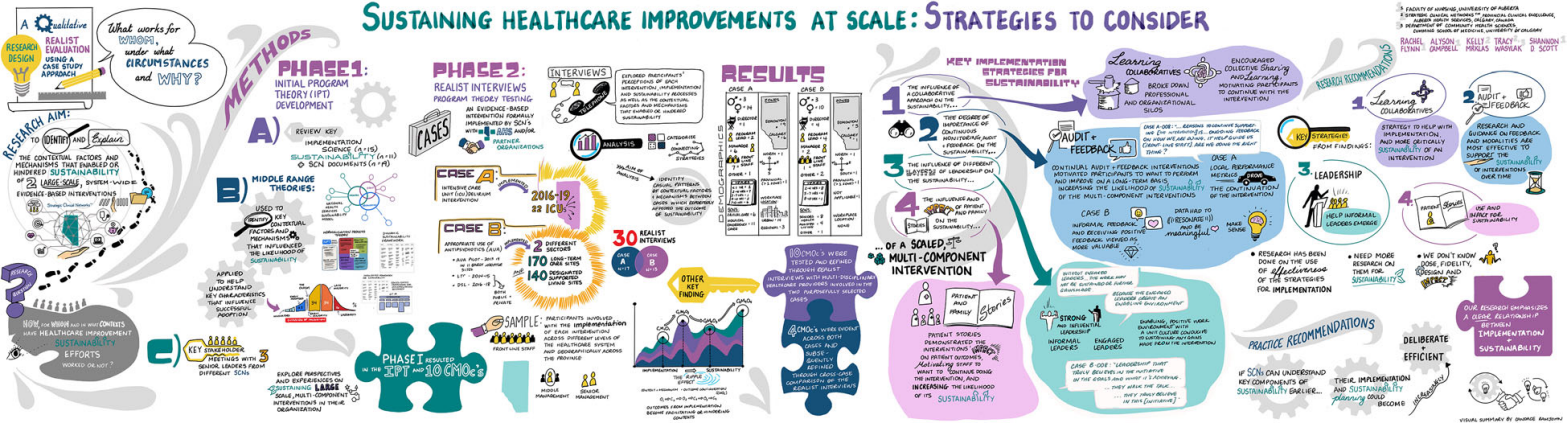
Visual summary of research process and findings. Developed by Candace Ramjohn at the Alberta SPOR SUPPORT Unit Learning Health System

Following a case study analysis approach [28], we analyzed case-specific CMOcs, followed by cross-case comparison of Case A and Case B CMOcs. It became clear during cross-case comparison analysis that similar CMOcs emerged across cases. Categorizing and connecting strategies outlined by Maxwell [42] were used to categorize CMOcs, with our IPT as an extraction guide. We also inductively coded new CMOcs that emerged across cases. We then connected CMOcs across cases using NVIVO 11 software. The aim of our analysis was to identify and explain contextual factors and causal mechanisms for the sustainability of both cases. Through cross case comparisons we could determine how the same causal mechanisms played out in different contexts and produced the same or different outcomes. In this paper, we report the most prominent CMOc patterns that emerged across both cases. See Fig. 3 for a visual summary of our research process and findings.

## Results

### Participant demographics

We conducted thirty realist interviews (case A, *n *= 17 and case B, *n *= 13) from July 2019 - October 2019. Participant demographics, by case, are presented in Table 2.

**Table 2:** Participant demographics by case

Variable	Case A (*n *= 17)	Case B (*n *= 13)
**Sex**		
Male	3	3
Female	14	10
**Role**		
Director	1	4
Program/Practice Lead	2	4
Manager (unit, program, patient-care)	6	2
Front-line Staff (physician, nurse, allied health)	7	1
Other (unspecified)	1	2
**Years in Role**		
Less than 1 year	1	-
2-4 years	7	8
5-7 years	1	4
8-10 years	2	1
10 years or more	6	-
**Workplace Setting**		
Critical Care SCN	6	-
Seniors Health SCN	-	8
Assisted/Facility Living	-	4
Hospital/Emergency Care	11	-
Other	-	1
**Workplace Zone**		
Edmonton	9	3
Calgary	6	1
North	1	1
South	-	1
Provincial (more than one zone)	1	6
Not applicable	**-**	1
**Workplace Location**		
Urban	14	**-**
Regional	3	**-**

### CMO configurations

From our initial ten CMOcs, three (CMOc 1, CMOc 2, CMOc 3) were evident across both cases and subsequently refined through cross-case comparison of the realist interviews. A fourth, novel CMOc (CMOc4) emerged across both cases that we had not hypothesized in our IPT. From our CMOc analysis we identified four important strategies to facilitating sustainability: Learning collaboratives, audit & feedback, the informal leadership role, and patient stories. These strategies triggered certain mechanisms such as sense-making, understanding value and impact of the intervention, empowerment, and motivation that increased the likelihood of sustainability within the context of two multi-component, scaled EBIs. We present our four CMOcs under the following headings: (1) *The influence of learning collaboratives on the sustainability of a scaled, multi-component intervention*; (2) *The degree of importance of continuous monitoring, audit and feedback on the sustainability of a scaled, multi-component intervention*, and (3) *The influence of informal leaders on the sustainability of a scaled, multi-component intervention*. A: (4) *The influence and impact of patient and family stories on the sustainability of a scaled, multi-component intervention.* These four CMOcs are presented in Table 3.

**Table 3:** CMOcs from realist interview findings

CMOc 1: The influence of learning collaboratives on the sustainability of a scaled, multi-component intervention	When an intervention is implemented at scale through a collaborative approach using a provincial learning collaborative that brings working groups, committees, and operational leaders across the province together (C), this break down existing silos (M), facilitates sharing among groups who otherwise may not interact (M), encourages cyclical reinforcement of the intervention (M) and facilitates discussions demonstrating the advantages and benefits of the intervention (O) this drives people to make the intervention a priority (M), encourages continuous learning, increasing the likelihood of intervention sustainability (O).
CMOc 2: The degree of importance of continuous monitoring, audit, and feedback on sustainability of a scaled, multi-component intervention	When an intervention is implemented at scale in a context where monitoring & feedback is done on a continual basis (C), through multiple communication and messaging channels (i.e. quality boards, staff meetings, emails) in a way that makes sense and resonates with different levels of staff (M), where staff can see unit performance, the extent of implementation effectiveness and observable benefits achieved (O), this triggers staff to have a better understanding of the extent of impact of the intervention (M), value unit performance, and motivates them to want to perform well and improve (M); this supports the continuation of the intervention and increases the likelihood of intervention sustainability (O).
CMOc 3: The influence of informal leaders on the sustainability of a scaled, multi-component intervention	When an intervention is implemented at scale in a context where strong and supportive leadership is present including front-line informal leaders (C), that show sustained interest in the intervention over time (M), are “hands on” and use their influence to positively communicate the impact and successes of the intervention (M), this triggers staff to pay more attention to the intervention, feel valued and empowered to use the intervention (Ms), where staff feel they are working in an environment conducive to sustaining gains made with the intervention (M) this supports the continuation of the intervention and increases the likelihood of intervention sustainability (O).
CMOc 4: The influence and impact of patient and family stories on the sustainability of a scaled, multi-component intervention	When an intervention is implemented provincially at scale (C) the use of patient or family stories to demonstrate the impact of the intervention to staff is powerful (M), patient stories trigger staff to understand the importance of the intervention and why it is needed (M) stories demonstrate the impact of the intervention for patient outcomes and improved care (O), this motivates staff to want to continue to do the intervention (M), increasing the likelihood of intervention sustainability (O)

### The influence of learning collaboratives on the sustainability of a scaled, multi-component intervention

*CMOc 1: When an intervention is implemented at scale through a collaborative approach using provincial learning collaboratives that brings working groups, committees, and operational leaders across the province together (C), this breaks down existing silos (M), facilitates sharing among groups who otherwise may not interact (M), encourages cyclical reinforcement of the intervention (M) and facilitates discussions demonstrating the advantages and benefits of the intervention (O) this drives people to make the intervention a priority (M), encourages continuous learning, increasing the likelihood of intervention sustainability (O).*

For both cases, an important context for sustainability was one where, in the early stages of implementation, a co-design approach was used to bring working groups, committees and operational leaders together provincially. A co-design approach was facilitated using provincial learning collaboratives (LCs), a strategy tailored to each case (see additional file 3 for LC case description).

 Participants reported that they felt that LCs broke down existing organizational and professional silos by bringing people together who may not otherwise interact, allowing them to discuss and share successes with the intervention. Participants felt that this space to share encouraged and motivated them to continue and sustain the work. LCs provided cyclical reinforcement of the intervention, continuous learning long-term.

Participants felt time constraints, financial and geographic barriers were major contextual hindrances to bringing people together, provincially for the LCs. For instance, in case B, front-line staff were not always able to attend every LC in person. In some instances, key staff were absent, due to the inability to secure time off or have shifts covered, insufficient budget to finance their attendance, or needing to travel long distances. To overcome these contextual barriers, LCs in case B were offered virtually. However, most front-line staff felt the value of LCs were bringing people together face-to-face. In contrast, directors and managers felt offering LCs virtually would be beneficial, especially considering anticipated future budget restraints, such as reduced staff travel funding. These participants considered virtual learning as a way to evolve, adapt and provide flexible learning in current fiscally restrained healthcare climates. It is unclear what, if any, impact differences there are in provincial “face-to-face” versus virtual LCs. Quotes to support this CMOc are presented in Table 4.

**Table 4:** Evidence to support CMOc1: the influence of learning collaboratives on the sustainability of a scaled, multi-component intervention

**Case A-002:** “So, every aspect of this intervention was collaborative and when I say that, the creation of it [the intervention] came from input and collaboration of operations, from units, to patients and families and to SCN staff. So, it was never done in silo of just a [name of SCN]. It was always done with an approach that there was representation from across the province.”
**Case A-009: “**We decided to use the innovative learning collaborative methodologies, which involved bringing together all 21 provincial teams, to be five learning sessions. And at these learning sessions, teams came together. We shared best practices. We shared guest presenters speaking about implementation. Speaking about clinical best practice for [name of intervention] and [name of work environment]. And teams had an opportunity to come together and network. They could work on…specific clinical best practices. There were four management metrics. And then they could choose two-unit specific metrics for which they chose best practices and clinical recommendations from the framework. And worked on implementing those through action plans of the learning collaboratives.”
**Case B-005**: “I think too another big piece was not having the intervention be just the responsibility of one person. So I think having, having the team actively engaged and involved and the team including families as well in that process. As we just talked earlier about the collaborative approach that you know, our medical director pitched in with the physicians. We had our program managers helping, coaching, mentoring the front line. You know our front-line nurses coaching and mentoring health care aides. So I think that was really key in that it wasn’t reliant on just one person to roll out the intervention that really, required a team effort and for everyone to be bought in. So I think that helped as well.”
**Case B-010**: “I really think it was the collaborative being an innovative collaborative. Having those three learning workshops. And the touch points in the middle, as opposed to having those one in done educations. Because you go to an education day, you get all hyped up, “oh my God! This is great information! We’re so excited!” And then you go back to your site and you are excited, but not all the other staff went to that education. And they have no idea what you are talking about. And then it is hard to implement something. Whereas when we do our collaboratives, we take a whole team. They come together and they make a plan on how to make change.”

### The degree of importance of continuous monitoring, audit, and feedback on sustainability of a scaled, multi-component intervention

***CMOc 2:*** *When an intervention is implemented at scale in a context where monitoring & feedback is done on a continual basis (C), through multiple communication and messaging channels (i.e. quality boards, staff meetings, emails) in a way that makes sense and resonates with different levels of staff (M), where staff can see unit performance, the extent of implementation effectiveness and observable benefits achieved (O), this triggers staff to have a better understanding of the extent of impact of the intervention (M), value unit performance, and motivates them to want to perform well and improve (M); this supports the continuation of the intervention and increases the likelihood of intervention sustainability (O).*

In both cases, the EBIs were implemented in contexts where strategies of continuous monitoring, audit, and feedback (A&F) of intervention data (i.e., provincial and local performance metrics, health outcomes, patient experiences) was shared with staff in ways that made sense and were meaningful. This context of continuous A&F of intervention data, such as provincial and local performance metrics, health outcomes and patient experiences triggered mechanisms of sense-making, understanding value of the intervention and motivation to continuously improve. Feedback was delivered to participants in each case, however different types of feedback were viewed as more important, depending on the intervention and stakeholders involved. Different stakeholders had different preferences and responses to the type of feedback that was meaningful to them.

In Case A, front-line staff felt that quantitative, provincial and local performance metrics “drove” continuation of the intervention. The continuous A&F of this data enabled staff *to have a better understanding of the extent of impact of the intervention.* Front-line staff felt this data allowed them to see and understand how they were performing in relation to other sites across the province, motivating them to continue to perform well and improve. Continuous A& F and subsequent mechanisms of sense-making and understanding the value and impact of the intervention supported the continuation of the intervention and increased the likelihood of sustainability. It is important to note that the dose of A&F delivered adapted over time, “monthly scorecards” providing local and provincial metrics, were provided at each site. After the initial implementation period of the intervention, quarterly performance metrics continued.

In contrast, for Case B, while the provincial and local performance metrics did hold some value in monitoring the intervention, participants felt it was especially important to consider contextual elements affecting these metrics. Sharing provincial and local performance metrics did not have the same impact. For instance, the purpose of Case B’s intervention was to reduce the *inappropriate* use of antipsychotics, rather than reduce all antipsychotics. Sometimes, leaving a resident on an antipsychotic was appropriate. Thus, staff felt that more specific data on *inappropriate antipsychotic use* and the use of alternative therapies (e.g., behavior therapy) was more valuable to see the impact of the intervention. For case B, the mode and delivery of A&F was through informal feedback, such as the sharing of success stories between sites and receiving positive feedback from families and other staff. Participants’ in Case B felt that informal feedback, through the sharing of success stories between sites, and receiving positive feedback from families and other staff, was more valuable intervention data. Importantly, all participants felt that the data being fed back had to resonate and be meaningful to its recipients and it was important for the data to “make-sense” to those reviewing it. Sense-making of data was viewed as a critical aspect of implementation that enabled sustainment.

Additionally, informal feedback, through the sharing of success stories between sites, and receiving positive feedback from families and other staff, was viewed as impactful. Staff in Case B felt informal feedback allowed them to see and understand the impact of the intervention and motivated them to continue.

Importantly, the way in which feedback was delivered to staff triggered different responses by staff which had an influence on sustainability. Multiple communication channels such as emails, scorecards, quality boards, and staff meetings were used. Participants made sense of, and responded to different communication channels, as different channels had different reach and impact. For example, front-line staff felt that emails were not an effective way to share data, because emails were often overlooked by front-line staff. However, managers and executive directors felt that email was often the most impactful way to share data as they were not overlooked. Quotes to support this CMOc are presented in Table 5.

**Table 5:** Evidence to support CMOc2: the degree of importance of continuous monitoring, audit, and feedback on sustainability of a scaled, multi-component intervention

**Case A-002:** “So, like the managers and the front-line staff who were part of these [name of intervention] committees, really valued how their units were performing. So really understanding what was happening every day. Not just what they think was happening. And there were often many times where it was like well I thought we were doing way better than that. And it was truly providing a very deeper understanding and insight into their daily unit practices. And that data was key in pushing this intervention forward and making those changes.”
**Case A-004**: “I think that is a huge driving factor [monitoring and feedback]. Because most people in [name of work environment], that is what drives them. If they know, okay this works, this is proven to work… this is the advantages. These are the pros and cons. This is why we need to make it [the intervention] a priority in our day.”
**Case A-008: “**Like I said, I think the reasons to continue supporting it [the intervention] is just because we do get this ongoing feedback on how we are doing. It helps guide us [front-line staff]. Are we doing the right thing? Are we doing the wrong thing? So, what do we have to change? And, you know, obviously seeing improvements in those metrics is motivating to continue doing those behaviors.”
**Case A-009: “**So I think that yes, the audit feedback is hugely important. But we have to be cognizant of peoples’ level of understanding. And also not overwhelming them. The way the data is presented is important so…if you’re presenting data to executive leadership for example and I’m thinking of executive directors, they may look at the data differently than a person at the front-line may look at the data. So they’re going to ask different questions. So I think presenting the data in a way that makes sense to the front-line staff.”
**Case B-002**: “There are so many new things coming at staff all the time that if you don’t keep referring back to results it just slides off people’s awareness. So, I think it is important that information continues to come back to sites whether that’s you know, in a quality board or in staff meetings or whatever. Otherwise it just disappears into the larger field of information that people see. So, I mean we’ve certainly had sites that have, you know, started out with really high levels of [name of clinical issue] that have dropped fairly dramatically. And then you look again, you know, six months or eight months later and their rates are rising again. So, I mean, it’s not just that the numbers are visible. It’s that somebody is actually looking at and them and giving some critical thought to why they’re doing what they’re doing. But I think if that information doesn’t keep coming back, you absolutely will not do that.”
**Case B-005**: “I think personally it’s very important because if we don’t measure and monitor, then how do you even know how you’re doing? So, I know that there’s been interventions in the past that we haven’t put monitoring mechanisms in place. Then it does just become flavor of the month and it kind of falls off the side of the plate. I think it’s important to remember that outside of [name of intervention], that there’s tons of interventions. So, I think it is super important to put in these mechanisms in place to ensure that we don’t get into that flavor of the month syndrome where it’s just dropped off peoples’ desk and it’s an afterthought. But if you’re continuously improving, you’re talking about it, you’re bringing forward the data, you’re having these conversations at meetings that it keeps it top of mind for folks.”

### The influence of informal leaders on the sustainability of a scaled, multi-component intervention

*CMOc3: When an intervention is implemented at scale in a context where strong and supportive leadership is present including front-line informal leaders (C), that show sustained interest in the intervention over time (M), are “hands on” and use their influence to positively communicate the impact and successes of the intervention (M), this triggers staff to pay more attention to the intervention, feel valued and empowered to use the intervention (Ms), where staff feel they are working in an environment conducive to sustaining gains made with the intervention (M) this supports the continuation of the intervention and increases the likelihood of intervention sustainability (O).*

Participants across both cases perceived strong and supportive leadership as an important strategy sustainability. Many participants reported that they felt strong and supportive leaders were not only managers or executive directors, but also front-line staff. Those considered to be informal leaders were “hands on”, showed an interest in the intervention, and are influential. Staff felt that influential informal leaders continually communicated the impact and successes of the intervention with others in a positive way. Positively communicating the impact and successes of the intervention with others encouraged staff to pay more attention to the intervention, and feel valued and empowered to use the intervention. Engaged informal leaders also created an enabling, positive work environment with a unit culture conducive to sustaining any gains made from the intervention. These contextual factors and mechanisms supported the sustainability of the intervention. Quotes to support this CMOc are presented in Table 6.

**Table 6:** Evidence to support CMOc3: the influence of informal leaders on the sustainability of a scaled, multi-component intervention

**Case A-009:** “So, the units that have been successful in, in their implementation have strong leadership endorsement for this work. And by leadership endorsement, I mean not just from an executive director position. That that trickles down. That comes from the unit managers who interact with the front-line staff on a daily basis. That comes from the patient care managers. And then the leadership as it goes up with [name of health service]. So, I think that having a strong leadership presence saying this work is important. Asking staff about it on a daily basis. So, having a conversation and that leadership doesn’t necessarily have to be even from the unit manager.”
**Case A-007**: “I think it [leadership] has to be somebody who has some sort of ability to make decisions and utilize resources. But also has a reasonable knowledge of how the front-line works. We often make [leaders] like our executive sponsors or our directors and such. And I don’t know that that’s the right way to do it. They’re very far removed from the actual work that’s being done.”
Case A-006: Well I think it [informal leaders] makes them [staff] excited about it [the intervention] and realize the importance [of the intervention]. And then when people see that it’s not just management that think this [the intervention] is a good thing and it’s a front-line person that understands it and thinks it’s important, people, I think, pay a little more attention.
**Case B-003: “**Well it’s pretty crystal clear to me without the engaged leaders, once the intervention ends, the work may not sustain, or further gains made. Because the engaged leaders create an enabling environment or develop an enabling environment for their front-line teams to work together. So, if you don’t have an enabling environment, this change just won’t happen.”
**Case B-008**: “I think leadership that truly believes in the intervention in the goals and what it’s achieving. I think leaders who are; they walk the talk. So… you know, they truly believe in this [intervention]. And I think too, leaders that are visible. Visible on the units. Visible again too depending on where the leader is in the organization…in terms of taking a look at the data. In terms of saying okay, let’s do the deeper dive. Let’s bring a group together to find out what’s happening, you know? So more hands on.”
**Case B-008**: Because if you have champions, then…most likely the reason why they’re champions is because they…they believe in this. They’re very passionate. And they have influence whether it be as a “formal” leader or informal leader. So…I think that [formal or informal leadership] helps to sustain [the intervention].

### The influence and impact of patient and family stories on the sustainability of a scaled, multi-component intervention

*CMOc4: When an intervention is implemented provincially at scale (C) the use of patient or family stories to demonstrate the impact of the intervention to staff is powerful (M), patient stories trigger staff to understand the importance of the intervention and why it is needed (M) stories demonstrate the impact of the intervention for patient outcomes and improved care (O), this motivates staff to want to continue to do the intervention (M), increasing the likelihood of intervention sustainability (O)*.

For participants from both cases, sharing a patient or family story was one of the most important strategies for sustainability in these contexts of scale across the province. In both cases, patient and family stories were formally shared as part of LCs, where everyone involved in the intervention participated provincially. Participants felt that a patient or family story helped them understand why the intervention was needed and the impact the intervention had on patient outcomes, which motivated staff to continue to sustain the intervention at scale. Some patient stories were shared in-person by family members, and some were shared in video format (digital stories). In Case B, stories were shared by family members of residents from sites across the province. In Case A, stories from patients and families across the province and publicly available videos (*delirium.org*) were shared. Participants felt they could really see and understand the importance of the intervention after hearing a patient or family story. Quotes to support this CMOc are presented in Table 7.

**Table 7:** Evidence to support CMOc4: the influence and impact of patient and family stories on the sustainability of a scaled, multi-component intervention

**Case A-002:** “So, we have five learning collaboratives. We always strive to have a patient and family story presented where we had a previous patient share their story with the audience of pictures and feedback and talking about what it felt like to be a patient. And our feedback that we received on that part of it was always very, very positive and that it was a patient story that really helped people to continue to push forward to make change and to continue with the work in terms of you know, I’ll say just continuing with our motivation to try. Because [name of clinical issue] is not a new practice in critical care and people often have said that they’re just you know, [name of clinical issue] fatigued. That they’re sick of hearing about it. They’re sick of doing the same kind of work and trying to make changes with it never happening. But one thing that we’ve heard loud and clear and continuing to hear is the patient story, really…I’ll say helped to overcome that fatigue.”
**Case A-009: “**So I think…and that’s been one of the most powerful things [patient and family stories]. A lot of people at the beginning said like this work is…not that they said it was dumb. But they said you know, “this is pointless. You’re never going to impact delirium. You’re not going to stop it. It’s still going to happen.” But once they saw the patient perspective…it really changed their motivation and why they wanted to do this work.”
**Case A-009**: “So it [patient story] created a lot of desire to make the change. Which was important because with all the different initiatives going on within AHS, people were really struggling saying you know, we have so many things going on right now, why should we work on [the intervention]? And once they heard the patient perspective or the effects and the outcomes of having delirium on their life once they were discharged from ICU, we had a lot more buy in from the staff.”
**Case A-013**: “Like when we first started doing delirium…we used a lot of the videos online…from the ICU delirum.org where there’s young people and the effects of their delirium on them and how it changed their long-term ability to manage was impactful actually for the staff”
**Case A-003**: “So one of the biggest things that I’ve seen through the learning sessions [collaboratives] and over the last two years, we did some of the family stories at the end. And that has a huge impact on the staff…because when you can identify with the families and the patients, it [intervention] seems to resonate when they can see how that’s impacted their lives.”
**Case B-005**: “So I think for buy-in [of the initiative] stories [from patients/families] definitely [helped with intervention buy-in]. I mean, data is one thing, but the stories are really what help people connect and, kind of have something to relate to. So I think that [stories] was one thing that when we saw that one story with that one person, we’re like “oh gosh”, you know? We [staff] really have an opportunity for improvement here and how can we do better?”
**Case B-009**: “So it [stories] connected people [staff] to the meaning and the purpose of their work. Where before it was very task focused. Once you understand that; once you experience that relational element of care and you have the meaning of bringing moments of happiness to people each day and you feel like you’re well supported by the team and the family members. And the family members are so pleased with the care. It’s positive feelings all, all around. So that’s, you know, part of the internal motivation [to sustain the work].”
**Case B-011**: “We got videos of teams talking about when a resident woke up. So you know, and we posted all of those stories on the toolkit so that people could use them and we talked about it as a strategy of using good news stories to encourage people and motivate them. So when health care aides say things like it’s actually easier to take care of people who can help then it was trying to take care of somebody who was so sedated that they couldn’t help themselves at all. That kind of became part of good news. But a lot of family stories about how I didn’t think I’d ever be able to talk to my dad again. And when he came off the anti-psychotics, we could have conversations. So that kind of thing became a really positive motivator for people continuing to do the work.“
**Case B-003**: “So the [case B education] package for front-line staff includes the success stories about Mrs. Jones who was on antipsychotics for a long period of time is now not. And, and while she was on anti-psychotics, you know, she was kind of drowsy and not participative or communicative. And now that we’ve been able to reduce or eliminate the use of antipsychotics, she’s up and about. So those success stories are what the front-line staff are most interested in. And families are interested in as well. Because that gives them [staff and family] the energy to continue to use behavioral approaches to managing…unwanted behaviors…instead of using pharmaceutical approaches to managing difficult behaviors.”

## Discussion

Our research findings explain important contextual factors, strategies and mechanisms that had a perceived effect on the sustainability of two provincially scaled, multi-component EBIs. Our discussion outlines four strategies viewed as critical to facilitating sustainability: Learning collaboratives, audit and feedback, informal leadership, and patient stories. These strategies produced common mechanisms of sense-making, understanding value and impact of the intervention, empowerment, and shared motivation which offer causal pathways explaining how these strategies achieved their desired outcomes. These mechanisms operated at the individual level, triggered by contextual factors and strategies at the unit and organizational level. It is important to note the macro context, that these were scaled interventions at a provincial level. This level of scale appeared to have facilitated a sense of shared understanding and motivation. To contribute to the knowledge gap of “how to sustain EBIs in healthcare” our discussion focuses on the four strategies introduced at implementation and causal mechanisms identified as pivotal to sustainability.

### Learning collaboratives as a strategy for sustainability

Collaborative research approaches are increasingly used by healthcare systems, research funders and government organizations as part of health services research and implementation science [43]. A collaborative research approach provides the opportunity for patients, healthcare providers and other key stakeholders to be active participants in the design process rather than the traditional approach of being a passive recipients of design work (i.e. intervention) [44]. Participants from both cases discussed LCs as a key implementation strategy that facilitated intervention sustainability. LCs encouraged participants to discuss and share successes and areas for improvement, leaving them feeling empowered and motivated to continue and sustain the work. In accordance with the Dynamic Sustainability Framework [26] our findings suggest that active partnership among all relevant stakeholders is essential to sustaining interventions within care settings. As in the Consolidated Framework for Sustainability [7], our research highlights the importance of relationships, collaboration, and networks for sustainability.

A LC is an organized, multifaceted approach that includes teams from multiple healthcare sites coming together to learn, apply and share improvement methods, ideas and data on performance for a given healthcare topic [45, 46]. In our evaluation, LCs occurred in-person for case A with virtual components introduced in case B. While there is clear evidence on the effectiveness of in-person LCs to enhance learning, less is known about the effectiveness of virtual LCs [47]. Similar to other research, our findings suggest that creating a culture of continuous learning, promoting accountability, and creating an inter-organizational support network from which sites can learn from others’ successes and challenges are some of the main benefits of LCs [48]. Our research identified how LCs triggered mechanisms of sharing among groups who otherwise may not interact, encouraged cyclical reinforcement of the intervention and facilitated discussions demonstrating the advantages and benefits of the intervention. These aspects drove people to make the intervention a priority, encouraged continuous learning, and increased the likelihood of intervention sustainability. Despite the benefits of LCs for sustainability identified in our study, and others, questions remain about the impact of LCs for sustained improvement and the and cost-analyses of LCs over time [46, 48, 49].

A systematic review by Wells et al., [46] found that LC characteristics, such as the number, length, and delivery mode (i.e. virtual vs. in-person) varied across studies. This highlights the existing variability in the design and delivery of LCs; there is a paucity of evidence on how best to design and implement a learning collaborative. Similar to Hoekstra et al., [43] we argue the need for research to examine how and why collaborative research approaches and interventions (such as LCs) work, including the key principles, strategies, outcomes, impacts and contextual conditions these approaches function under. This knowledge may allow for more tailored and efficient stakeholder engagement in future.

### Continuous monitoring, audit, and feedback for sustained change

Monitoring, audit, and feedback (A&F) of interventions are important strategies to facilitate buy-in, maintain compliance and ensure the continuation of improved outcomes [50]. Our findings pertaining to how A&F supports ongoing staff engagement, by hearing, and seeing data in a group atmosphere are well aligned with the literature [50–52].

The use of data to monitor local implementation is not just a means of promoting accountability, but also a strategy to solve problems that impair performance. In the absence of regular, careful monitoring, implementation may be more liable to fail or revert to previous practices [50]. From our findings, it is evident that careful and continuous monitoring, A&F needs to happen from early implementation of an intervention to support sustainability. Implementation teams and operational leaders need to plan a monitoring, A&F system that makes sense and is meaningful to all of those involved and can demonstrate impact.

Previous research has been done to synthesize the effectiveness of A&F for implementation research. One Cochrane systematic review on 140 studies found that A&F can lead to important improvements in professional practice. However, the effectiveness of A&F as an intervention to change provider behavior depends on both the content of and how the feedback is provided [51]. The Dynamic Sustainability Framework [26] suggests that ongoing feedback on interventions should use practical, important measures of progress and relevance. The framework recommends the use of measures that are feasible, relevant to desired outcomes of patients and align with the ‘fit’ between intervention and context. There is a lack of guidance on what dose of feedback and which modalities are most effective to support the sustainability of scaled interventions over time. A&F is most effective when provided more than once [51], however it is unclear from the literature and our study, how often the intervention is required for sustainable impact. Another study that examined the use of theory in A&F studies found that there was an overall lack of use and consistency of explicit theory to guide A&F interventions [52]. As a result of these issues, the most important active ingredients and mechanisms that enable successful A&F intervention for healthcare improvement remain unclear [53]. Our findings identified that continuous A&F triggered mechanisms of staff to have a better understanding of the extent of impact of the intervention, value unit performance, and motivated them to want to perform well and improve. Which lead to the continuation of the intervention and increased the likelihood of intervention sustainability.

In an effort to bridge this knowledge gap, Ivers et al., [53] provided potential best practice guidance recommendations for A&F interventions in relation to audit components, feedback components, the nature of behavior change required and target, goals and action plan. Taking study findings into account, we concur with these best practice recommendations. Our results further emphasize the presence of variance in contextual factors (e.g., resource allocation), intervention design (e.g., mode of delivery of feedback, frequency of feedback,), recipient characteristics (e.g., profession, role, years of experience) and behavior change characteristics (e.g. readiness for change, practice change) that influence the effect of A&F on sustainability. Future research is needed to examine the process of delivery, effectiveness, and impact of A&F on the sustainability of multi-component, scaled interventions, even in a single provincial system undertaking coordinated, provincial implementation and scale.

### The influence of informal leadership for sustainability

Previous implementation research has established the influence of formal (e.g., administrators) and informal leaders (e.g., champions) and their activities (e.g., facilitation, support) on sustainability [1, 54, 55]. Informal leaders, sometimes referred to as champions, opinion leaders, change agents, or knowledge brokers, are considered front-line practitioners, driving the implementation of a wide range of change interventions in healthcare settings [56–58].

A focus on informal leaders is essential because this is where the quality of care ultimately affects patient outcomes [59]. In alignment with our study, a Cochrane review determined that the effectiveness of informal leaders as a strategy for the implementation of evidence-based interventions appears comparable, or sometimes even superior, to other interventions [60]. As in our study, Ennis et al., [61] found that informal leaders contribute to creating a positive work environment. Informal leaders influence workplace culture and have significant impacts on team efficacy and performance by seeking out opportunities to promote, improve and negotiate best care practices [61].

Our findings suggest that front-line informal leaders are valued and play an important role in the implementation and sustainability of multi-component, scaled interventions. In our study, front-line informal leaders were active participants in the intervention and were encouraging and motivating for others. This aligns with existing evidence that informal leaders are effective because they socially influence other professionals, and that this influence is a function of the respect of their peers [58, 60]. Furthermore, it was recognized that senior leaders (i.e. executive directors, unit managers) may not necessarily be the best people to promote continuation of interventions due to their lack of understanding of the daily work of front-line staff. Informal leaders were viewed as more influential based on their credibility amongst colleagues. This same phenomenon has been found in similar work [62].

Engaging influential individuals across organizations can help to secure the credibility of interventions and strategies to develop “informal leaders” have shown to be effective in implementing changes at the clinical level [62]. Hence, implementation strategies should recognize and seek to engage with and develop individuals who have not traditionally been perceived as leaders. In the later stages of implementation, senior leadership should plan for strategies to help informal leaders emerge, ensuring they have the capacity and capabilities to lead in sustaining efforts. Like the Consolidated Framework for Sustainability Constructs in Healthcare [7] our research highlights the importance of the people involved (e.g., champions) for sustainability.

### Impact of sharing patient and family stories

In our initial program theory, we did not hypothesize patient stories as an important strategy for the sustainability of an intervention. Patient stories have previously shown merit, with reported improvements in care practices, positive staff engagement, a way for staff to “remember why we’re here”, and combat burnout [63, 64]. In our study, patient stories provided a way for participants to connect with patients, understand their experiences, and remind them why the intervention was important, motivating them to sustain the work.

Patient stories have a degree of emotional power that can spark attention, resonance and change [65–68]. Our study, and others, have found that sharing patient success stories enables HCPs to feel energized after watching them, as these stories are “impactful, heartwarming, and understandable” [64]. Foster et al.,[69] found that listening to patient stories not only had profound emotional effects on HCPs, but motivated practice change as they developed newly formed intentions to improve patient outcomes. Similarly, Haigh and Hardy Haigh and Hardy [70] found that patient stories shown to HCPs led to reflection, empathy and discussions surrounding practice change aimed at service improvement. These studies mirror our findings in that sharing patient stories can influence better service and patient outcomes through staff motivation and reflection of current practice. Our research identified that patient stories were a powerful strategy to demonstrate the impact of the intervention to staff. Patient stories triggered staff to understand the importance of the intervention and why it is needed. Patient stories demonstrated the impact of the intervention for patient outcomes and improved care, which triggered mechanisms of motivation whereby staff wanted to continue to do the intervention. This led to the increased likelihood of intervention sustainability.

Despite the clear impact our study, and others, have shown of patient stories on staff motivation, it is less clear how these stories are being used, to what end they are collected, and how often they need to be shared to sustain initial levels of motivation [64].

### Research and practice implications

Our findings found four key strategies (use of collaborative approach, A&F, informal leadership, and patient stories) perceived by participants to positively influence intervention sustainability. Importantly, from these key strategies, we identified causal mechanisms of sustainability, notably sense-making, understanding value and impact of the intervention, and shared motivation. Understanding and explaining these mechanisms is crucial to ensure that selected strategies are tailored to target identified implementation and sustainability determinants. Without this careful a-priori planning, it is possible the “wrong” strategy will be implemented, whereby the strategies are not tailored to specific contexts, negatively impacting sustainability [12] and long-term impact.

Our research also highlighted knowledge gaps that require further research. There is a lack of rigorous evaluations on the use and effectiveness of LCs as a strategy to sustain impact and improvement. More research needs to be done to look at the design, components, delivery, and impact of LCs as a strategy to help with sustainability of an intervention. For A&F further research is needed to evaluate different approaches to the design, delivery, and dose of this intervention for sustainability outcomes. We also recommend research that can unpack and try to explain theory used in A&F design and effect modifiers of A&F. Lessons from such research can help researchers and decision- makers plan, design and execute improvement interventions in a way that can be done before implementation and that can lead to sustainable outcomes and impact. Our research recommends that senior leadership needs to plan for strategies to help informal leaders to emerge and to ensure that they have the capacity and capabilities to lead intervention implementation and sustainability efforts. Patient stories have been identified as powerful strategy to translate knowledge, however evaluations are needed in relation to the use and impact of patient stories for sustainability.

Our work also aligns with and extends existing theoretical approaches for sustainability. For example, the Consolidated Framework for Sustainability presents 40 determinants that influence the sustainability of healthcare interventions, such as leadership and champions, monitoring progress over time, stakeholder participation and involvement [7]. Our research offers potential strategies (i.e. learning collaboratives, A&F, and patient stories) to increase the likelihood of intervention sustainability and impact. Understanding how to sustain scaled interventions, through which strategies is a novel area in sustainability research. We recommend future research that tests the effectiveness and validity of these strategies for sustainability across other scaled interventions.

Resource allocation is challenging in health systems, thus it is important for implementers to understand what they ‘need to do’ vs. ‘what is nice to do’ in order to create and maintain interventions that have sustainable impact. Our research has shown that learning collaboratives, A&F, informal leaders and shared patient stories have a perceived positive influence on sustainability; yet it remains unknown which of these strategies are a ‘need to do’ versus a ‘nice to do’ for long-term sustainability and impact. There is also a clear tension between implementation and sustainability, it is unclear for operational leaders how much effort to put into sustainability planning prior to implementation when it is unknown if an intervention will be successful or not. Nonetheless, our research emphasizes a clear relationship between implementation and sustainability; we anticipate that if SCNs can understand key components of sustainability earlier, their implementation and sustainability planning could become increasingly deliberate and efficient. Our findings illustrated how implementation of two multi-component EBIs at scale (target change at organisational/system level) created conducive contexts and triggered positive mechanisms for change at other levels (e.g. individual behaviour change). Our research also demonstrates how the design of implementation strategies intended to facilitate sustainability processes and outcomes needs to consider and be tailored to identified determinants of sustainability and contextual factors. The four key strategies identified in our evaluation triggered positive mechanisms of sense-making, understanding value and impact of the intervention, empowerment, and motivation that will increase the likelihood of sustained EBIs in practice. These findings have practice implications for future SCN implementation efforts at scale and elements that need to considered.

### Limitations

The strategies and mechanisms identified in this evaluation are based on the perceptions of our participants from two scaled interventions; additional research is needed to test the influence of these factors on sustainability outcomes, *in situ*, and among other scaled interventions. It was beyond the scope of this study to examine the sustainment of the interventions in terms of impact on clinical outcomes. To mitigate this limitation, we purposely sought out several data sources (SCN leaders, documents, including theory and existing evidence to inform the link between implementation and sustainability, participant interviews) to inform our work across all stages of the research. Our sampling of individuals within each intervention attempted to access those who could best reflect on intervention implementation and sustainability. During our Case B interviews, we learned emergently that health care aides may be a key informant role that we had not yet accessed. We subsequently attempted, but were unsuccessful at recruiting individuals to participate in study interviews, and this may have negatively impacted our ability to fully characterize unique aspects of that intervention in our study.

## Conclusions

To date many implementation science evaluations (e.g., process evaluations) have identified determinants (barriers and facilitators) to successful implementation (uptake) and sustainability of an intervention. Great work has been done on strategies for implementation and sustainability, and identified outcomes for implementation and sustainability, but we have yet to truly unpack the causal mechanisms at interplay between these factors to lead to sustained and impactful change. Our findings provide important lessons and considerations for other scaled interventions and healthcare systems looking to adopt and sustain scaled, multi-component evidence-based interventions. We identified four key strategies (i.e., learning collaboratives, audit and feedback, informal leaders, and patient stories) and subsequent mechanisms that enabled the likelihood of sustainability. Future research that tests these strategies for sustainability can help to provide evidence-based recommendations to healthcare innovators, leaders, researchers, and decision-makers on how to optimize impact of interventions by thinking of sustainability from the outset. Until such research is done, scarce healthcare resources will continue to be wasted on interventions that cannot be sustained.

## Supplementary Information

**Additional file 1.** Case and intervention descriptions.

**Additional file 2. **RAMESES II reporting standards for realist evaluations.

**Additional file 3. **Standards for Quality Improvement Reporting Excellence (SQUIRE 2.0).

**Additional file 4. **Initial program theory development: CMOc mapping and hypotheses.

## Data Availability

The qualitative data supporting this study is not available as participants did not consent to having their data publicly available but anonymized quotations are available from the corresponding author on reasonable request.

## Abbreviations

EBIs: Evidence-based interventions
SCNs: Strategic Clinical Networks™
AHS: Alberta Health Services
ICU: Intensive Care Unit
AUA: Appropriate Use of Antipsychotics
LTC: Long-term care
DSL: Designated supportive living
IPT: Initial program theory
CMOc: Context-Mechanism-Outcome Configuration
LCs: Learning collaboratives
A&F: Audit and feedback
