# Supplementary material for: Contextual factors and mechanisms that influence sustainability: a realist evaluation of two scaled, multi-component interventions
Source: BMC Health Serv Res. 2021 Nov 4;21:1194. doi: 10.1186/s12913-021-07214-5 (PMC8570000; doi:10.1186/s12913-021-07214-5)
Supplement: Supplementary file 1 — Additional file 1. Case and intervention descriptions. [file 12913_2021_7214_MOESM1_ESM.docx]

**Additional File 1: Case and intervention descriptions**

**Case A: Delirium intervention**

Case A is a provincially scaled Intensive Care Unit (ICU) multi-component intervention aimed to a) decrease the incidence and duration of ICU delirium, b) reduce ICU length of stay / hospital length of stay; c) improve quality of ICU delirium care across Alberta using evidence-informed best practices; d) collaborate & learn together as a critical care community and; e) provide access to ICU delirium resources for patients, families and healthcare providers across Alberta.

This intervention is led by Alberta Health Services’ (AHS) Critical Care Strategic Clinical Network™ (CC SCN). The delirium intervention was underpinned by two main sources of empirical evidence: 1) The ABCDEF (**A**ssess, prevent and manage pain; **B**oth Spontaneous Awakening Trials (SAT) and Spontaneous Breaking Trials (SBT); **C**hoice of analgesia and sedation; **D**elirium: assess, prevent and manage; **E**arly mobility and exercise; **F**amily engagement and empowerment) care bundle of best practices to prevent and manage ICU delirium and 2) the Pain, Agitation, and Delirium (PAD) clinical practice guidelines (1) which were updated in 2018 (2)

Several implementation science strategies were used to implement the delirium intervention at scale (i.e., collaborative design, audit & feedback, implementation champions and formally appointed implementation leaders, among others) at all 22 ICUs across Alberta. Province-wide implementation occurred from September 2016 to March 2019 by operational and medical leaders and frontline teams in 22 ICUs across the 16 major hospital sites in AHS.

Implementation of the delirium intervention included the use of an AHS adaptation of the IHI Innovative Learning Collaborative (ILC) approach (3) to engage local teams in using measurement to drive clinical pathway practice changes and achieve system-wide improvements. This approach allowed for co-design of the plan for implementation and selection of relevant measures (selected from the ABCDEF Bundle) and capacity building for both formal and informal leaders. These ILCs included learning sessions where interprofessional teams from each of the 22 ICUs, patients/families, and expert faculty exchanged ideas and developed specific action plans to achieve targets. Balanced Scorecards were used to organize key provincial and locally identified indicators to audit and feedback data for six different dimensions of quality (Safety, Effectiveness, Efficiency, Appropriateness, Accessibility, and Acceptability). A total of five ILCs occurred during Provincial ICU Delirium intervention implementation, involving an average of 173 multi-disciplinary participants from across Alberta ICUs.

Multi-disciplinary implementation teams comprised frontline health professionals and operational leaders. Each local ICU team was supported by Practice Leads that led implementation activities to facilitate higher adoption rates and sustainable practices. Monthly audit and feedback reports were provided to frontline providers and unit leaders; quarterly feedback reports were provided to hospital site and zone leadership. A provincial dashboard of the spread and scale of the ABCDEF bundles was monitored by the SCN and reported to Senior Leadership within AHS. All 22 units monitored elements of the bundle (based on baseline data of their performance) and over the course of the project showed marked improvements in reducing incidence of delirium (10% reduction), reductions in both ICU and Hospital LOS, increased screening of delirium risk (40% to 75%), increased standardization of evidence based practices in every ICU across the province (4). More information on the delirium intervention can be accessed at: <https://albertahealthservices.ca/scns/Page13415.aspx>

**Case B: Appropriate Use of Antipsychotics (AUA) medications intervention**

The Appropriate Use of Antipsychotics (AUA) medications intervention for supportive living (SL) and long-term care (LTC) was developed and implemented by the Alberta Health Services (AHS) Seniors Health Strategic Clinical Network™ (SCN). The aim of the AUA intervention was to reduce the inappropriate use of antipsychotic medications for managing responsive behaviors associated with dementia. Risks and side-effects of antipsychotic medications include agitation, confusion, falls, insomnia, and sedation, along with increased risk of infection, strokes, and cardiac events. The AUA project is aimed at helping staff enhance care for persons with dementia by focusing on person-centered approaches. Care teams considered each person's unique life story, looked for underlying reasons for agitation and in lieu of prescribing medications looked to address these needs. A dementia-friendly approach requires families, physicians, and staff to work together to investigate and trial alternative approaches to reduce agitation and anxiety.

The AUA intervention was first implemented in 11 “early adopter” long term care (LTC) sites across Alberta in 2013-2014. The intervention was designed to support long term care staff to review and intervene on those clients whose antipsychotic medication was no longer warranted. To accomplish this, the Seniors Health SCN created and implemented a provincial AUA guideline <https://extranet.ahsnet.ca/teams/policydocuments/1/clp-ahs-appropriate-use-antipsychotic-med-ltc-guideline-ps-26-01.pdf> and a toolkit with a bundle of evidence-based resources <https://www.albertahealthservices.ca/scns/auatoolkit.aspx> were created and customized to local sites. An inter-professional monthly antipsychotic medication review was one of the key strategies of the AUA intervention. Following the success of these early adopter sites in reducing antipsychotic use by 50%, the intervention was then scaled and spread across all 170 LTC facilitates across Alberta in 2014-2015 (both AHS & privately owned and operated facilities), followed by implementation in 165 designated supportive living (DSL) facilities from 2016-2018.

The AUA intervention also utilized an AHS-adaptation of the IHI Innovative Learning Collaborative (ILC) approach. The aim of this approach is to integrate effective evidence-based implementation strategies through collaborative design (learning and action periods, iterative audit-feedback cycles to facilitate dynamic implementation, use evidence-based strategies to address arising barriers/facilitators and to measure clinical pathway practice change, champions and formally appointed implementation leaders, efforts to assess practice compatibility and sustainability, among others) (3). The ILC method, tools and training were standardized to achieve: 1) care engagement; 2) focus on quality and 3) finish to sustainment (5). The AUA implementation process also included staff education to create informal champions, discussions with family members (to ensure patient /family stories and lived experience are part of the process), and development of resident-specific care plans. A monthly inter-professional medication review was implemented to track, assesses, and reduces inappropriate use of these medications. Strategies to facilitate uptake of the AUA intervention also included learning workshops, monthly curbside consultation call-in sessions, video conferences and the AUA toolkit of resources.

After successful implementation of three ILCs in 11 early adopter sites, a series of nine ILCs (3 full-day sessions for each collaborative) were conducted to facilitate provincial scaled implementation in 2014-2015. Sessions engaged over 100 LTC sites with higher than provincial average baseline rates of resident antipsychotic medication use. Between 8-18 sites participated in each collaborative (one “change team” from each site). These “change-team” members were responsible for attending workshops, educating staff at their site, organizing and supporting practice change, and collecting data for the AUA intervention team. Seven of nine collaboratives occurred in person and two occurred virtually. The remaining LTC sites (approximately 60) were invited to one 2-hour educational session about the AUA toolkit and resources. These sites had lower than provincial average use of antipsychotics in their residents and received education only. In 2016-2018, with the spread to DSL, a series of 4 learning workshops engaged 156 DSL sites. Learning workshops occurred face-to-face, with some sites joining virtually. A total of 69 learning workshop sessions were held from 2016-2018.

Additionally, informed by a refined implementation strategy gleaned from addressing emergent barriers and facilitators, the AUA intervention team provided 11 videoconferences, and monthly curbside consultations with the intent of supporting implementation teams in the field. Curbside consultations involved teleconference meetings to discuss case studies on responsive behaviors of residents with dementia. These additional meetings were opportunities for collaborative learning, problem solving, and support. In DSL sites, Antipsychotic use in Alberta LTC has decreased by more than 30% in the past 4.5 years. Just 17.1% of Alberta’s LTC residents (without a chronic mental health condition) are using antipsychotic medications, compared to the national average of 21.2% (2017-18). More information on the AUA intervention can be accessed at: <https://www.albertahealthservices.ca/scns/auatoolkit.aspx>

References

1. Barr J, Fraser GL, Puntillo K, Ely EW, GÉLinas C, Dasta JF, et al. Clinical practice guidelines for the management of pain, agitation, and delirium in adult patients in the intensive care unit. Critical Care Medicine. 2013;70(41):263-306.

2. Devlin JW, Skrobik Y, Gélinas C, Needham DM, Slooter AJC, Pandharipande PP, et al. Clinical Practice Guidelines for the Prevention and Management of Pain, Agitation/Sedation, Delirium, Immobility, and Sleep Disruption in Adult Patients in the ICU. Critical Care Medicine. 2018;46(9):1532-48.

3. Institute for Healthcare Improvement. The Breakthrough Series: IHI’s collaborative model for achieving breakthrough improvement. 2003.

4. Alberta Health Services. Alberta's Strategic Clinical Networks Improving Health Outcomes: Retrospective 2012-2018.; 2019.

5. Arain M, Charland P, Graham L, Memon A, Ahmad A. Appropriate Use of Antipsychotics in Designated Supportive Living 4/4D Evaluation: Final Report. Calgary, AB: Health Systems Evaluation and Evidence Alberta Health Services; 2018.
