# Supplementary material for: Contextual factors and mechanisms that influence sustainability: a realist evaluation of two scaled, multi-component interventions
Source: BMC Health Serv Res. 2021 Nov 4;21:1194. doi: 10.1186/s12913-021-07214-5 (PMC8570000; doi:10.1186/s12913-021-07214-5)
Supplement: Supplementary file 2 — Additional file 2. RAMESES II reporting standards for realist evaluations. [file 12913_2021_7214_MOESM2_ESM.docx]

| **Additional File 2.** RAMESES II reporting standards for realist evaluations. | | |
| --- | --- | --- |
| **List of items to be included when reporting realist evaluations** | **Reported in document Y/N/Unclear** | **Page(s) in document** |
| **1.Title**  identify the document as a realist evaluation | Contextual factors and mechanisms that influence sustainability: A realist evaluation of two scaled, multi-component interventions |  |
| **2. Summary of abstract**  Journal articles will usually require an abstract, while reports and other forms of publication will usually benefit from a short summary. The abstract or summary should include brief details on: the policy, programme or initiative under evaluation; programme setting; purpose of the evaluation; evaluation question(s) and/or objective(s); evaluation strategy; data collection, documentation and analysis methods; key findings and conclusions Where journals require it and the nature of the study is appropriate, brief details of respondents to the evaluation and recruitment and sampling processes may also be included Sufficient detail should be provided to identify that a realist approach was used and that realist programme theory was developed and/or refined | In our abstract we describe details on:  The two scaled, multi-component interventions under evaluation: a delirium intervention for Critical Care and an Appropriate Use of Antipsychotics (AUA) intervention for Senior’s Health.  Purpose of the evaluation: to identify and explain the contextual factors and mechanisms perceived to influence the sustainability of two provincial SCN evidence-based interventions | Page 1 |
| **Introduction**  **3. Rationale for evaluation**  Explain the purpose of the evaluation and the implications for its focus and design  **4. Programme theory**  Describe the initial programme theory (or theories) that underpin the programme, policy or initiative  **5. Evaluation questions, objectives and focus**  State the evaluation question(s) and specify the objectives for the evaluation. Describe whether and how the programme theory was used to define the scope and focus of the evaluation  **6. Ethical approval**  State whether the realist evaluation required and has gained ethical approval from the relevant authorities, providing details as appropriate. If ethical approval was deemed unnecessary, explain why | 3. Rationale for evaluation: The aim of our study was to identify and explain the contextual factors and mechanisms that enabled or hindered the sustainability of two, large-scale, system-wide evidence-based interventions implemented across the Strategic Clinical Networks™, of the Alberta health system in Canada .No studies to date have explicitly examined sustainability of large-scale SCN interventions. Recognizing and explaining key factors that have hindered and facilitated SCN intervention sustainability will contribute to systematic and comprehensive sustainability planning. This realist evaluation case study examines two multi-component interventions that have been spread and scaled across Alberta, providing an opportunity to better understand contextual factors and mechanisms that influence sustainability at scale. We purposefully selected two scaled, evidence-based multi-component interventions based on a) their maturity, b) scale of implementation (province wide), c) demonstration of improved outcomes and impact and, d) context variation (community and acute healthcare). We defined a ‘case’ as an intervention that was evidence-based, had been formally implemented by the SCNs either within Alberta Health Services and /or with partner organizations.  4. Programme theory: The first step in our IPT was to review key implementation science, sustainability and SCN documents. Various theories, models and frameworks were used to identify key contextual factors and mechanisms that influenced the likelihood of sustainability and to help understand key characteristics that influence successful intervention adoption. Second, we conducted key stakeholder meetings with senior leaders from different SCNs, to explore their perspectives and experiences on the sustainability of scaled interventions.  5. Evaluation questions, objectives and focus: The objective of our realist evaluation was to study factors that enabled or hindered the sustainability of two provincially scaled and spread multi-component interventions or “cases” by examining “what works for whom, under what circumstances, and why?”, rather than merely assessing “does it work?”.  6. Ethics approval for this study was granted by the University of Alberta Health Research Ethics Board (Pro0096202). Institutional approval was provided by Alberta Health Services Northern Alberta Clinical Trials and Research Centre. | Page 4-5  Page 6,  Additional File 4  Page 5-6  Page 5 |
| **Methods**  **7 Rationale for using realist evaluation**  Explain why a realist evaluation approach was chosen and (if relevant) adapted  **8 Environment surrounding the evaluation**  Describe the environment in which the evaluation took place  **9 Describe the programme policy, initiative or product evaluated**  Provide relevant details on the programme, policy or initiative evaluated  **10 Describe and justify the evaluation design**  A description and justification of the evaluation design (i.e. the account of what was planned, done and why) should be included, at least in summary form or as an appendix, in the document which presents the main findings. If this is not done, the omission should be justified and a reference or link to the evaluation design given. It may also be useful to publish or make freely available (e.g. online on a website) any original evaluation design document or protocol, where they exist  **11 Data collection methods**  Describe and justify the data collection methods – which ones were used, why and how they fed into developing, supporting, refuting or refining programme theory Provide details of the steps taken to enhance the trustworthiness of data collection and documentation  **12 Recruitment process and sampling strategy** Describe how respondents to the evaluation were recruited or engaged and how the sample contributed to the development, support, refutation or refinement of programme theory  **13 Data analysis**  Describe in detail how data were analysed. This section should include information on the constructs that were identified, the process of analysis, how the programme theory was further developed, supported, refuted and refined, and (where relevant) how analysis changed as the evaluation unfolded | 7. Rationale for realist evaluation: Realist evaluation unpacks and explains the possible causes and contextual factors of change by examining “what works for whom, under what circumstances, and why?”, rather than merely assessing “does it work?”. We followed the realist heuristic context (C) + mechanism (M) = outcome (O) configuration, whereby an intervention works or not (O), (CMOcs) because of the action of some underlying mechanism (M), which only comes into operation in particular contexts (C). We followed the realist cycle of theory hypothesis generation, theory hypothesis observation and specification.  8. Environment surrounding evaluation: Our evaluation took place within two provincially scaled and spread multi-component interventions “cases”. Case A is the Intensive Care Unit (ICU) Delirium intervention implemented from 2016-19 across all 22 ICUs in Alberta. Case B is the Appropriate Use of Antipsychotics (AUA) implemented in two different sectors, long-term care (LTC, 170 sites) and designated supportive living (DSL, 140 sites). The AUA intervention was first piloted in 2013-14 in 11 early adopter sites and was spread provincially during 2014-15 to 170 LTC sites (both public and private); DSL implementation occurred from 2016-18 in 140 spaces both public and private settings (see additional file 3 for case descriptions).  9. Describe the program policy, initiative or product evaluated: We fully describe both interventions in additional file 3.  10. Describe and justify evaluation design: We conducted a realist evaluation using an explanatory case study research design. We followed the realist heuristic context (C) + mechanism (M) = outcome (O) configuration, whereby an intervention works or not (O), (CMOcs) because of the action of some underlying mechanism (M), which only comes into operation in particular contexts (C). We followed the realist cycle of theory hypothesis generation, theory hypothesis observation and specification.  11. Data collection methods: We conducted qualitative realist interviews using a semi-structured interview guide to test and further refine our initial program theory and explore new emerging CMOcs. All interviews were conducted by telephone by the research assistant, audio recorded and transcribed.  12. Recruitment process and sampling strategy: We purposefully selected interview participants involved with the implementation of each intervention across different levels of the healthcare system (i.e., front line staff, middle management, and senior management) and geographically across the province. We contacted potential study participants through an open letter of invitation circulated to staff by SCN leaders. Interested participants were invited to voluntarily contact the research assistant at their convenience for more information.  13. Data analysis: Following a case study analysis approach (1), we analyzed case-specific CMOcs, followed by cross-case comparison of Case A and Case B CMOcs. It became clear during cross-case comparison analysis that similar patterns emerged across cases. Categorizing and connecting strategies outlined by Maxwell (2) were used to categorize CMO patterns, with our IPT as an extraction guide. We also inductively coded new CMOcs that emerged across cases. We then connected CMO patterns across cases using NVIVO 11 software to code CMOc patterns. | Page 5  Page 7  Additional File 3  Page 4-5.  Page 7  Page 7  Page 8 |
| **Results**  **14 Details of participants**  Report (if applicable) who took part in the evaluation, the details of the data they provided and how the data was used to develop, support, refute or refine programme theory  **15 Main findings**  Present the key findings, linking them to contexts, mechanisms, and outcome configurations. Show how they were used to further develop, test or refine the programme theory | 14. Details of participants: See table 1  15. Main findings: Our main findings include from our initial ten CMOcs, three that were evident across both cases and subsequently refined through cross-case comparison of the realist interviews. A fourth, novel CMOc emerged across both cases that we had not hypothesized in our IPT.  We provide evidence to support each CMOc in Tables 3-6. | Table 1  Page 8-17, Tables 3-6 |
| **Discussion**  **16 Summary of findings**  Summarise the main findings with attention to the evaluation questions, purpose of the evaluation, programme theory and intended audience  **17 Strengths, limitations and future directions** Discuss both the strengths of the evaluation and its limitations. These should include (but need not be limited to): (1) consideration of all the steps in the evaluation processes; and (2) comment on the adequacy, trustworthiness and value of the explanatory insights which emerged In many evaluations, there will be an expectation to provide guidance on future directions for the programme, policy or initiative, its implementation and/or design. The particular implications arising from the realist nature of the findings should be reflected in these discussions  **18 Comparison with existing literature**  Where appropriate, compare and contrast the evaluation’s findings with the existing literature on similar programmes, policies or initiatives | Discussion:  16. Summary of findings: Our findings provide important lessons and considerations for other scaled interventions and healthcare systems looking to adopt and sustain scaled, multi-component evidence-based interventions. We identified four key strategies (i.e., learning collaboratives, audit and feedback, informal leaders, and patient stories) that enabled the likelihood of sustainability.  17. Strengths, limitations, future directions: We describe strengths, limitations and future directions throughout our discussion.  18. Comparison with existing literature: We incorporate comparisons with existing literature throughout the discussion. | Page 17-22  Page 23  Page 22 |
| **19 Conclusion and recommendations**  List the main conclusions that are justified by the analyses of the data. If appropriate, offer recommendations consistent with a realist approach | Our findings provide important lessons and considerations for other scaled interventions and healthcare systems looking to adopt and sustain scaled, multi-component evidence-based interventions. We identified four key strategies (i.e., learning collaboratives, audit and feedback, informal leaders, and patient stories) that enabled the likelihood of sustainability. | Page 23 |
| **20 Funding and conflict of interest**  State the funding source (if any) for the evaluation, the role played by the funder (if any) and any conflicts of interests of the evaluator | No conflicts of interest to report and a funding statement is provided. | Page 24 |
