# Supplementary material for: Contextual factors and mechanisms that influence sustainability: a realist evaluation of two scaled, multi-component interventions
Source: BMC Health Serv Res. 2021 Nov 4;21:1194. doi: 10.1186/s12913-021-07214-5 (PMC8570000; doi:10.1186/s12913-021-07214-5)
Supplement: Supplementary file 3 — Additional file 3. Standards for Quality Improvement Reporting Excellence (SQUIRE 2.0). [file 12913_2021_7214_MOESM3_ESM.docx]

**Additional File 3. Standards for Quality Improvement Reporting Excellence (SQUIRE 2.0)**

| **Text Section and Item**  **Name** | **Section or Item Description** | **Page Number** |
| --- | --- | --- |
| **Title and Abstract** |  |  |
| **1. Title** | Indicate that the manuscript concerns an [initiative](#_bookmark6) to improve healthcare (broadly defined to include the quality, safety, effectiveness, patient- centeredness, timeliness, cost, efficiency, and equity of healthcare) | Title page |
| **2. Abstract** | 1. Provide adequate information to aid in searching and indexing 2. Summarize all key information from various sections of the text using the abstract format of the intended publication or a structured summary such as: background, local [problem,](#_bookmark10) methods, interventions,   results, conclusions | Page 1 |
| **Introduction** | *Why did you start?* |  |
| [**3. Problem**](#_bookmark10) **Description** | Nature and significance of the local [problem](#_bookmark10) | Page 3-4 ‘Research Context: Strategic Clinical Networks, Alberta Health Services’ |
| **4. Available knowledge** | Summary of what is currently known about the [problem,](#_bookmark10) including relevant previous studies | Page 3-4 ‘Research Context: Strategic Clinical Networks, Alberta Health Services’  Additional file 3 |
| **5. Rationale** | Informal or formal frameworks, models, concepts, and/or [theories](#_bookmark14) used to explain the [problem,](#_bookmark10) any reasons or [assumptions](#_bookmark0) that were used to develop the [intervention(s),](#_bookmark8) and reasons why the [intervention(s)](#_bookmark8) was  expected to work | Page 5‘Realist evaluation’  Page 5-6 ‘Initial program theory development’ |
| **6. Specific aims** | Purpose of the project and of this report | Page 2 ‘Research aim’ |
| **Methods** | *What did you do?* |  |
| **7.** [**Context**](#_bookmark1) | Contextual elements considered important at the outset of introducing the [intervention(s)](#_bookmark8) | Page 4 ‘Research Context: Strategic Clinical Networks, Alberta Health Services’ |
| **8.** [**Intervention(s)**](#_bookmark8) | 1. Description of the [intervention(s)](#_bookmark8) in sufficient detail that others could reproduce it   Specifics of the team involved in the work | Additional File 1. Case descriptions |
| **9. Study of the Intervention(s)** | 1. Approach chosen for assessing the impact of the [intervention(s)](#_bookmark8)   Approach used to establish whether the observed outcomes were due to the [intervention(s)](#_bookmark8) | Page 4 ‘Realist evaluation’  Page 7 ‘Recruitment and data collection’ |
| **10. Measures** | 1. Measures chosen for studying [processes](#_bookmark11) and outcomes of the [intervention(s),](#_bookmark8) including rationale for choosing them, their operational definitions, and their validity and reliability 2. Description of the approach to the ongoing assessment of contextual elements that contributed to the success, failure, efficiency, and cost 3. Methods employed for assessing completeness and accuracy of data | Page 4 ‘Realist evaluation’  Page 5-6 ‘Initial program theory development’  Page 7  ‘Recruitment and data collection’ |
| **11. Analysis** | 1. Qualitative and quantitative methods used to draw [inferences](#_bookmark5) from the data 2. Methods for understanding variation within the data, including the 3. effects of time as a variable | Page 7  ‘Recruitment and data collection’ |
| **12. Ethical**  **Considerations** | [Ethical aspects](#_bookmark2) of implementing and studying the [intervention(s)](#_bookmark8) and how they were addressed, including, but not limited to, formal ethics review and potential conflict(s) of interest | Page 6 ‘Ethics’ |
| **Results** | *What did you find?* |  |
| **13. Results** | 1. Initial steps of the [intervention(s)](#_bookmark8) and their evolution over time (*e.g.*, time-line diagram, flow chart, or table), including modifications made to the intervention during the project 2. Details of the [process](#_bookmark11) measures and outcome 3. Contextual elements that interacted with the [intervention(s)](#_bookmark8) 4. Observed associations between outcomes, interventions, and relevant contextual elements 5. Unintended consequences such as unexpected benefits, problems, failures, or costs associated with the [intervention(s).](#_bookmark8)   Details about missing data | Page 8 -14 ‘CMO configurations’  Tables 2-6 participant quotes |
| **Discussion** | *What does it mean?* |  |
| **14. Summary** | 1. Key findings, including relevance to the [rationale](#_bookmark12) and specific aims   Particular strengths of the project | Page 14-21 |
| **15. Interpretation** | 1. Nature of the association between the [intervention(s)](#_bookmark8) and the outcomes 2. Comparison of results with findings from other publications 3. Impact of the project on people and [systems](#_bookmark13) 4. Reasons for any differences between observed and anticipated outcomes, including the influence of [context](#_bookmark1)   Costs and strategic trade-offs, including [opportunity costs](#_bookmark9) | Page 14-21 |
| **16. Limitations** | 1. Limits to the [generalizability](#_bookmark3) of the work 2. Factors that might have limited [internal validity](#_bookmark7) such as confounding, bias, or imprecision in the design, methods, measurement, or analysis   Efforts made to minimize and adjust for limitations | Page 22 |
| **17. Conclusions** | 1. Usefulness of the work 2. Sustainability 3. Potential for spread to other [contexts](#_bookmark1) 4. Implications for practice and for further study in the field 5. Suggested next steps | Page 23 |
| **Other information** |  |  |
| **18. Funding** | Sources of funding that supported this work. Role, if any, of the funding organization in the design, implementation, interpretation, and reporting | Page 24 |
