# Supplementary material for: Contextual factors and mechanisms that influence sustainability: a realist evaluation of two scaled, multi-component interventions
Source: BMC Health Serv Res. 2021 Nov 4;21:1194. doi: 10.1186/s12913-021-07214-5 (PMC8570000; doi:10.1186/s12913-021-07214-5)
Supplement: Supplementary file 4 — Additional file 4. Initial program theory development: CMOc mapping and hypotheses. [file 12913_2021_7214_MOESM4_ESM.docx]

| **Additional File 4. Initial program theory development: CMOc mapping and hypotheses** | | | | |
| --- | --- | --- | --- | --- |
| **Theme: Linkage between assessment of barriers and facilitators with sustainability**  **CMOc hypothesis 1:** If an intervention has been implemented within a context where barriers and facilitators to successful uptake have been assessed (C) and implementation is designed and adjusted for using theory driven, evidence-based implementation strategies (M), then the intervention is more likely to be tailored to the needs of the end user (O), is more likely to be adopted (O), may increase readiness for change and may be more likely to be sustained (O). | | | | |
| **Context (C1)** | **Mechanism (M1)** | **Outcome (O1)** | **Link to formal theory** | **Reference** |
| If an intervention has been implemented within a context where barriers and facilitators to successful uptake have been assessed | Implementation is designed and adjusted for using theory driven, evidence-based implementation strategies | The intervention is:  More likely to be tailored to the needs of the end user  More likely to be adopted  And  May increase readiness for change  May be more likely to be sustained | TDF: There is recognition that interventions to change behavior should draw on theories of behavior and behavior change. Mapping barriers and facilitators to behavior change techniques is a way to use theory to optimize the benefits of theory-based interventions. Their appropriate application depends on mapping techniques onto proposed behavioural determinants (barriers/facilitators).; this is critical for the development and reporting of effective interventions.  CFIR: CFIR specifies a list of constructs within general domains that are believed to influence (positively or negatively, as specified) implementation (and possibly sustainability). CFIR is a meta-theoretical framework that is built from other theories that contain references to sustainability (e.g., REAIM, PRISM etc.).  Using CFIR helps researchers assess the extent to which implementation is effective in a specific setting, prolongs sustainability and promotes dissemination. | Michie S, Johnston M, Francis J, Hardeman W, Eccles M. From Theory to Intervention: Mapping Theoretically Derived Behavioural Determinants to Behaviour Change Techniques. Theory-Based Health Behavior Change. 2008(4):660  Cane J, O'Connor D, Michie S. Validation of the theoretical domains framework for use in behaviour change and implementation research. Implementation Science. 2012;7(1):37-53.  Damschroder LJ, Aron DC, Rosalind KE, Kirsh SR, Alexander JA, Lowery JC. Fostering implementation of health services research findings into practice: a consolidated framework for advancing implementation science. Implementation Science. 2009; 4(1):50.doi: 10.1186/1748-5908-4-50.  French, S. D., Green, S. E., O’Connor, D. A., McKenzie, J. E., Francis, J. J., Michie, S., … Grimshaw, J. M. (2012). Developing theory-informed behaviour change interventions to implement evidence into practice: a systematic approach using the Theoretical Domains Framework. *Implementation Science*, *7*(1), 38–45. https://doi.org/10.1186/1748-5908-7-38 |
| **Theme: Addressing sustainability prior to implementation**  **CMOc hypothesis 2:** If an intervention is implemented in a context where sustainability has been considered by all stakeholders involved in planning, implementing, leading or receiving the intervention (e.g., unit managers, frontline staff, physicians, intervention managers, senior consultants, Senior Provincial Director, researcher) prior to and as part of implementation (C), then the intervention is more likely to be designed in a way that is better tailored to the setting (O), where stakeholders feel that they can “choose” the “active ingredients” adapt and own the intervention to fit with their own environment and workflow (M), stakeholders can make-sense of the intervention within the context of their own work, they feel empowered to be involved in the intervention and understand what is needed to sustain the intervention (M), and take collective action to embed the new intervention in practice (M), this creates a long-term shared vision and environment for sustained change, where the intervention becomes routine daily practice (O). | | | | |
| **Context (C2)** | **Mechanism (M2)** | **Outcome (O2)** | **Link to formal theory** | **Reference** |
| Context where sustainability has been considered by all stakeholders involved in planning, implementing, leading or receiving the intervention prior to and as part of the implementation | Stakeholders feel that they can “choose” the “active ingredients” adapt and own the intervention to fit with their own environment and workflow  Stakeholders can make-sense of the intervention within the context of their own work, they feel empowered to act and understand what is needed to sustain the intervention and take collective action to embed the new intervention in practice | The intervention is more likely to be designed in a way that is better tailored to the setting.  This creates a long-term shared vision and environment for sustained change, where the intervention becomes routine daily practice | Normalization Process Theory - Coherence, cognitive participation and collective action | May C, Finch T, Mair F, et al. Understanding the implementation of complex interventions in health care: the normalization process model. BMC Health Serv Res. 2007;7(1):148-154.<https://doi.org/10.1186/1472-6963-7-148> |
| **Theme: The influence of a collaborative approach on the sustainability of the intervention**  **CMOc hypothesis 3:** If an intervention is implemented using a collaborative approach* (C) then those impacted by implementation of the intervention are more likely to have considered explicitly their/others roles in making change happen prior to implementation (M), and the intervention is more likely to be adopted (O), stakeholders are more likely to feel ownership of the intervention (M), the intervention is more likely to have been trialed within the implementation environment with barriers/facilitators to uptake anticipated (O), and the intervention is more likely to be sustained (O). | | | | |
| **Context (C3)**  Context where the intervention is implemented using a collaborative approach | **Mechanism (M3)**  Those impacted by implementation of the intervention are more likely to have considered explicitly their/others roles in making change happen prior to implementation  Stakeholders are more likely to feel ownership of the intervention | **Outcome (O3)**  The intervention is more likely to be adopted  The intervention is more likely to have been trialed within the implementation environment and barriers/facilitators to uptake anticipated  The intervention is more likely to be sustained | **Link to formal theories**  Characteristics of IKT and participatory research processes (Graham et al, 2014)  NHS SM: Staff Factor 5: Staff involvement and training to sustain the process: Whether staff play a part in the implementation of changes to processes and the extent of training and development of staff to help sustain these changes  Staff Factor 6: Staff attitudes towards sustaining the change: Whether staff ideas are taken on board, the opportunity they are given to test these ideas and their belief that this is a better way of doing things that should be preserved. | **References**  Graham, Tetroe, Pearson (Eds) 2014. Turning Knowledge into Action: Practical Guidance on how to do integrated KT research.  May C, Finch T, Mair F, et al. Understanding the implementation of complex interventions in health care: the normalization process model. BMC Health Serv Res. 2007;7(1):148-154.<https://doi.org/10.1186/1472-6963-7-148> |
| **Theme: Linking individual values to the sustainability of the intervention**  **CMOc hypothesis 4 :** If the intervention is implemented in a context where there is shared value by all involved (C), the intervention makes sense (M), and has produced observable benefits to not only patients (efficiency, workload)(O), this triggers staff to believe in the intervention, feel engaged in the change process and become involved in the intervention (M) which will then make staff more likely to adopt and integrate the new practice as a routine part of care, leading to the intervention being sustained in practice (O). | | | | |
| **Context (C4)** | **Mechanism (M4)** | **Outcome (O4)** | **Link to formal theories** | **Reference** |
| Context where there is shared value by all involved | The intervention makes sense  Staff believe in the intervention, feel engaged in the change process and become involved in the intervention | The intervention produces observable benefits to not only patients (efficiency, workload)  Staff adopt and integrate the new practice as a routine part of care, leading to the intervention being sustained in practice | Links to DOI relative advantage and observability  Links to NPT coherence  Links to NHS SM process factor 1 & 2 | May C, Finch T, Mair F, et al. Understanding the implementation of complex interventions in health care: the normalization process model. BMC Health Serv Res. 2007;7(1):148-154.<https://doi.org/10.1186/1472-6963-7-148>  Maher L, Gustafson D, Evans A. NHS Sustainability Model. NHS Institute for Innovation and Improvement; 2010. [http://webarchive.nationalarchives.gov.uk/20160805122935/http://www.nhsiq.nhs.uk/media/2757778/nhs_sustainability_model_-_february_2010_1_.pdf](http://webarchive.nationalarchives.gov.uk/20160805122935/http:/www.nhsiq.nhs.uk/media/2757778/nhs_sustainability_model_-_february_2010_1_.pdf).  Rogers, Everett M. *Diffusion of Innovations.* 5th ed., Free Press trade pbk. ed. New York: Free Press, 2003. |
| **Theme: The influence of relationships amongst various stakeholders on sustainability**  **CMOc hypothesis 5:** If the intervention is implemented in a context where there are good existing relationships amongst various stakeholder groups and capacity to make change (C), where key individuals remain in the health economy (even if their roles and job titles change) and interpersonal relationships remain “warm,” (C) this triggers stakeholders to feel that there is a shared sense of values, priorities and needs (Ms), where stakeholders feel safe and empowered to trial the intervention and learn from it (M), this creates a unit culture conducive to improvement, where these priorities, needs and solutions are continually negotiated and adapted to aid sustainability and utility of the intervention can be optimized (O). | | | | |
| **Context (C5)** | **Mechanism (M5)** | **Outcome (O5)** | **Link to formal theories** | **Reference** |
| Context where there are good existing relationships amongst various stakeholder groups and capacity to make change  Context where key individuals remain in the health economy (even if their roles and job titles change) and interpersonal relationships remain “warm,” | Stakeholder groups feel a shared sense of values, priorities and needs  Stakeholder groups feel safe and empowered to trial the intervention and learn from it | A unit culture conducive to improvement, where these priorities, needs and solutions are continually negotiated and adapted to aid sustainability and utility of the intervention can be optimized. | DSF Framework argues that partnerships among all relevant stakeholders are essential to maintaining and improving interventions within care settings.  NHS SM Process Factor 3: Adaptability of improved processes | Chambers DA, Glasgow RE, Stange KC. The dynamic sustainability framework: addressing the paradox of sustainment amid ongoing change. Implementation Science: IS. 2013;8:117.  Maher L, Gustafson D, Evans A. NHS Sustainability Model. NHS Institute for Innovation and Improvement; 2010. [http://webarchive.nationalarchives.gov.uk/20160805122935/http://www.nhsiq.nhs.uk/media/2757778/nhs_sustainability_model_-_february_2010_1_.pdf](http://webarchive.nationalarchives.gov.uk/20160805122935/http:/www.nhsiq.nhs.uk/media/2757778/nhs_sustainability_model_-_february_2010_1_.pdf). |
| **Theme: The influence of adaptation on sustainability**  **CMOc hypothesis 6**: In a healthcare context that is complex and dynamic (C), mutual adaptations must be made between the intervention and the organizations current state to meet demands (political, organizational, environmental, financial, professional and patient), this triggers the ability to respond to and embrace change and the belief that an intervention can optimally be utilized (M), which will improve fit and alignment of the intervention (O) and adjust for demands (e.g., new integration of practices, new evidence, priority changes or resource availability), this embraces change as a central influence on sustainability and creates a culture of ongoing improvement, continual reflection and customization of interventions to current demands (O). | | | | |
| **Context (C6)** | **Mechanism (M6)** | **Outcome (O6)** | **Link to formal theories** | **Reference** |
| Context that is complex and dynamic | Mutual adaptations must be made between the intervention and the organizations current state to meet demands (political, organizational, environmental, financial, professional and patient)  Ability to respond to and embrace change and the belief that an intervention can optimally be utilized | Improves fit and alignment of the intervention and adjusts for demands (e.g., new integration of practices, new evidence, priority changes or resource availability)  embraces change as a central influence on sustainability and creates a culture of ongoing improvement, continual reflection and customization of interventions to current demands | DSF Framework  embraces change as  a central influence on sustainability. Adaptation is expected,  and even encouraged.  NHS SM - Process factor 3: adaptability of improved processes | Chambers DA, Glasgow RE, Stange KC. The dynamic sustainability framework: addressing the paradox of sustainment amid ongoing change. Implementation Science: IS. 2013;8:117. |
| **Theme: The degree of importance of continuous monitoring, evaluation, and feedback on sustainability**  **CMOc hypothesis 7:** If the intervention is being used in a context where continuous monitoring and evaluation is embedded in daily work (C), and fed back in a way that makes sense to stakeholders (M), this enables people to understand the extent to which implementation was effective and delivered as intended or adapted to meet demands (M), and what observable benefits have been achieved (O), this triggers people to feel motivated to continue to use the intervention (M), and provides those involved with the intervention with the ability to determine and respond to essential sustainability factors (M), to continually improve the intervention, promoting sustainment of the intervention where it is more likely to become routinized, adopted into everyday culture and norms of an organization (O). | | | | |
| **Context (C7)** | **Mechanism (M7)** | **Outcome (O7)** | **Link to formal theories** | **Reference** |
| Context where continuous monitoring and evaluation is embedded in daily work, | Feedback of results in a way that makes sense to stakeholders  Enables people to understand the extent to which implementation is effective and delivered as intended or adapted to meet demands  People feel motivated to continue use of the intervention  Provides those involved with the intervention the ability to determine and respond to the factors that are essential to sustain the intervention | Observable benefits have been achieved  Continually improve the intervention  Promoting sustainment of the intervention where it is more likely to become routinized, adopted into everyday culture and norms of an organization | DSF- Ongoing feedback on interventions should use practical,  relevant measures of progress and relevance  DSF - Assessment of care settings and outcomes is ongoing and incorporated within practice,  and staffing and policy changes are incorporated in sustainability  planning.  DSF- Interventions can be continually improved, boosting sustainment in practice, and can enable ongoing learning among developers, interventionists, researchers and patients  The DSF suggests that optimal fit requires that characteristics of the intervention, practice setting, and ecological system be consistently tracked, using valid, reliable and relevant measures, and expects that interventions, settings and the ecological system should change over  time, particularly where data can suggest improvements for each to better meet the needs of patients,  CFS: How the intervention design and delivery influences sustainability, in particular constructs of monitoring progress over time  NHS SM- Process Factor 4: Effectiveness of the system to monitor progress: Whether data are easily available to monitor progress or assess improvement and whether there are systems to communicate this in the organization. | Chambers DA, Glasgow RE, Stange KC. The dynamic sustainability framework: addressing the paradox of sustainment amid ongoing change. Implementation Science: IS. 2013;8:117.  Maher L, Gustafson D, Evans A. NHS Sustainability Model. NHS Institute for Innovation and Improvement; 2010. [http://webarchive.nationalarchives.gov.uk/20160805122935/http://www.nhsiq.nhs.uk/media/2757778/nhs_sustainability_model_-_february_2010_1_.pdf](http://webarchive.nationalarchives.gov.uk/20160805122935/http:/www.nhsiq.nhs.uk/media/2757778/nhs_sustainability_model_-_february_2010_1_.pdf).  Lennox L, Maher L, Reed J. Navigating the sustainability landscape: a systematic review of sustainability approaches in healthcare. Implementation Science. 2018; 13:27. |
| **Theme: Influence of stakeholder involvement on sustainability**  **CMOc hypothesis 8:** If an intervention is implemented in a context where stakeholders are continuously engaged and involved throughout the planning, implementation and adaptation processes (C), where stakeholders are able to share their ideas regularly, and have been able to access and receive training on the intervention (M), this should help increase the fit between the intervention and the local context (O) where stakeholders feel more confident in working with the intervention (M), this will help address evolving issues that might interfere with sustainability (O) and trigger the belief that the intervention is a better way of doing things (O) and form partnerships among all relevant stakeholders that are essential to maintaining and improving interventions within care settings over time (O). | | | | |
| **Context (C8)** | **Mechanism (M8)** | **Outcome (O8)** | **Link to formal theories** | **Reference** |
| Context where stakeholders are continuously engaged and involved throughout the planning, implementation and adaptation processes | Where stakeholders are able to share their ideas regularly, and have been able to access and receive training on the intervention    Stakeholders feel more confident in working with the intervention    Belief that the intervention is a better way of doing things | Increase the fit between the intervention and the local context  Address evolving issues that might interfere with sustainability and trigger the  The intervention is more likely to be sustained overtime. | NHS SM- Staff factors 6, 7 & 8  DSF - Ongoing stakeholder involvement throughout should  lead to better sustainability  CFS: How people involved are important to sustainability, in particular the constructs of stakeholder participation, staff and patient involvement. | Chambers DA, Glasgow RE, Stange KC. The dynamic sustainability framework: addressing the paradox of sustainment amid ongoing change. Implementation Science 2013; 8:117.  Maher L, Gustafson D, Evans A. NHS Sustainability Model. NHS Institute for Innovation and Improvement; 2010. [http://webarchive.nationalarchives.gov.uk/20160805122935/http://www.nhsiq.nhs.uk/media/2757778/nhs_sustainability_model_-_february_2010_1_.pdf](http://webarchive.nationalarchives.gov.uk/20160805122935/http:/www.nhsiq.nhs.uk/media/2757778/nhs_sustainability_model_-_february_2010_1_.pdf).  Lennox L, Maher L, Reed J. Navigating the sustainability landscape: a systematic review of sustainability approaches in healthcare. Implementation Science. 2018; 13:27. |
| **Theme: Influence of different layers of leadership**  **CMOc hypothesis 9:** If an intervention is implemented in a context where credible and respected leaders are present at various levels (C) and leaders are seen promoting and investing their own time in the intervention (M), where leaders are highly involved and visible in their support of the change process (C), and use their influence to communicate the impact of the intervention and break down barriers (M) then staff are more likely to have trust and believe in the intervention (O), regularly share information with and actively seek advice from leaders about the intervention (O), creating a change culture that is conducive of adopting, implementing and sustaining the intervention (O). | | | | |
| **Context (C9)** | **Mechanism (M9)** | **Outcome (O9)** | **Link to formal theories** | **Reference** |
| Context where credible and respected leaders are present at various levels and are seen promoting and investing their own time in the intervention | Leaders are seen promoting and investing their own time in the intervention  Leaders are highly involved and visible in their support of the change process  Use their influence to communicate the impact of the intervention and break down barriers | Staff are more likely to have trust and believe in the intervention  Regularly share information with and actively seek advice from leaders about the intervention  Creating a change culture that is conducive of adopting, implementing and sustaining the intervention | NHS Sustainability Model- Staff factor 7 and 8 | Maher L, Gustafson D, Evans A. NHS Sustainability Model. NHS Institute for Innovation and Improvement; 2010. [http://webarchive.nationalarchives.gov.uk/20160805122935/http://www.nhsiq.nhs.uk/media/2757778/nhs_sustainability_model_-_february_2010_1_.pdf](http://webarchive.nationalarchives.gov.uk/20160805122935/http:/www.nhsiq.nhs.uk/media/2757778/nhs_sustainability_model_-_february_2010_1_.pdf). |
| **Theme: Influence of intervention “fit” within the organization**  **CMOc hypothesis 10:** In a context where attention to “fit” between the intervention and the implementation setting is continually assessed, where the intervention aligns with the organization’s strategic aims and culture and the organization has adequate infrastructure for sustainability (staff, facilities, equipment and policies and procedures) (C), then staff can be trained in the new way of working and feel more confident in carrying out the intervention (M) and are more likely to feel as though the intervention is relevant to them and their work (M) which should improve sustainment and identify opportunities for intervention improvement (O). | | | | |
| **Context (C10)** | **Mechanism (M10)** | **Outcome (O10)** | **Link to formal theories** | **Reference** |
| Context where attention to “fit” between the intervention and the implementation setting is continually assessed, where the intervention aligns with the organization’s strategic aims and culture and the organization has adequate infrastructure for sustainability (staff, facilities, equipment and policies and procedures) | Staff can be trained in the new way of working and feel more confident in carrying out the intervention  More likely to feel as though the intervention is relevant to them and their work | Should improve sustainment and identify opportunities for intervention improvement | NHS Sustainability Model- Organization factors 9 and 10  DSF- Programs should be more likely to be maintained when there is strong ‘fit” between the program and the implementation setting  Specific to the DSF, is the expectation that change is constant at  each of these levels (and ripples across multiple levels), and thus the success of an intervention to be sustained over time lies in the measured, negotiated, and reciprocal fit of an intervention within a practice setting and the practice setting within the larger ecological system. | Chambers DA, Glasgow RE, Stange KC. The dynamic sustainability framework: addressing the paradox of sustainment amid ongoing change. Implementation Science 2013; 8:117.  Maher L, Gustafson D, Evans A. NHS Sustainability Model. NHS Institute for Innovation and Improvement; 2010. [http://webarchive.nationalarchives.gov.uk/20160805122935/http://www.nhsiq.nhs.uk/media/2757778/nhs_sustainability_model_-_february_2010_1_.pdf](http://webarchive.nationalarchives.gov.uk/20160805122935/http:/www.nhsiq.nhs.uk/media/2757778/nhs_sustainability_model_-_february_2010_1_.pdf).  Lennox L, Maher L, Reed J. Navigating the sustainability landscape: a systematic review of sustainability approaches in healthcare. Implementation Science. 2018; 13:27. |
| TDF Theoretical Domains Framework; CFIR Consolidated Framework for Implementation Research; DSF Dynamic Sustainability Framework, NHS SM National Health Service Sustainability Model; CFS Consolidated Framework for Sustainability; DOI Diffusion of Innovations; NPT Normalization Process Theory; IKT Integrated Knowledge Translation; RE-AIM The Reach-Efficacy-Adoption-Implementation-Maintenance Framework; PRISM The Practical Robust Implementation and Sustainability Model. | | | | |
